# Stepwise mitochondria‐targeted photodynamic immunotherapy enabled by an outer membrane vesicles‐based nanoplatform for synergistic tumor ablation and immune reprogramming

**DOI:** 10.1002/smo2.70039

**Published:** 2026-02-19

**Authors:** Xiang Cheng, Ziheng Luo, Ying Yin, Duoyang Fan, Ruyan Xie, Yanpeng Fang, Xiaohui Liu, Dayan Xiong, Wei Liu, Seraphine V. Wegner, Fei Chen, Wenbin Zeng

**Affiliations:** ^1^ Xiangya School of Pharmaceutical Sciences Central South University Changsha Hunan China; ^2^ Hunan Key Laboratory of Diagnostic and Therapeutic Drug Research for Chronic Diseases Changsha China; ^3^ Sun Yat‐Sen Memorial Hospital Sun Yat‐Sen University Guangzhou China; ^4^ The Affiliated Nanhua Hospital Hengyang Medical School University of South China Hengyang China; ^5^ Xiangya School of Nursing Central South University Changsha Hunan China; ^6^ Institute of Physiological Chemistry and Pathobiochemistry University of Münster Münster Germany

**Keywords:** bacterial outer membrane vesicles, macrophage polarization, photodynamic therapy, photosensitizers

## Abstract

Photodynamic immunotherapy (PDIT) integrates reactive oxygen species (ROS)‐mediated tumor destruction with immune activation. However, its effectiveness is often hindered by tumor hypoxia, poor tumor‐targeted delivery, and the immunosuppressive microenvironment. Here, we introduce **BDPM@OMVs**, a biomimetic nanoplatform designed to overcome these challenges. This system uses bacterial outer membrane vesicles to encapsulate a novel, heavy‐atom‐free aggregation‐induced emission photosensitizer (**BDPM**). Our platform enables stepwise lysosome‐to‐mitochondria trafficking for enhanced PDIT. **BDPM@OMVs** exhibits strong near‐infrared absorption and efficient intersystem crossing, leading to both Type I and Type II ROS generation, which sustains photodynamic performance even under hypoxic conditions. Upon light irradiation, **BDPM@OMVs** trigger photochemical internalization (PCI), disrupting lysosomes and releasing **BDPM** into the cytosol. The freed **BDPM** then selectively accumulates in mitochondria, where continues light exposure generates robust ROS, causing mitochondrial dysfunction and activating apoptosis. This process effectively amplifies tumor cell eradication. Simultaneously, **BDPM@OMVs** reprogram the tumor immune microenvironment by promoting macrophage repolarization from the immunosuppressive M2 to the pro‐inflammatory M1 phenotype, as evidenced by upregulated TNF‐α, IL‐1β, and CD86 expression. In vivo studies confirm that **BDPM@OMVs** achieve efficient tumor accumulation, allow for real‐time NIR imaging, and provide superior therapeutic outcomes. This work presents a versatile and hypoxia‐resilient PDIT strategy that synergistically integrates precise subcellular photodamage with immune modulation to overcome resistance in solid tumors.

## Graphical abstract

We constructed a novel excellent photodynamic drug without heavy atoms based on the conventional BODIPY photosensitizer, and then obtained a photodynamic vesicle with efficient tumor targeting (**BDPM@OMVs**) by bacterial extravasation. Multiple reactive oxygen species were efficiently generated under light irradiation, and the spontaneous elimination of **BDPM@OMVs** guided photointernalization to induce mitochondrial damage and apoptosis in tumor cells. The resulting debris further excites macrophages to stimulate the induction of anti‐tumor immune responses. Thus **BDPM@OMVs**‐mediated therapy efficiently eradicates solid tumors through enhanced photodynamic immunotherapy.

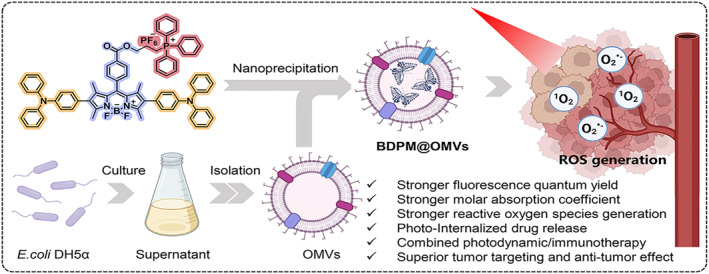

## 1 INTRODUCTION

Immunotherapy suppresses tumor growth, metastasis, and recurrence by directly activating or restoring antitumor immune responses, and has fundamentally transformed clinical cancer treatment.^[1]^ As an attractive immunotherapeutic strategy, cancer vaccines aim to elicit antigen‐specific immune responses through the delivery of tumor‐associated antigens.^[2]^ However, the major limitation of these approaches is the significant variability in individual immune responsiveness, which can compromise treatment outcomes. In contrast, photodynamically enhanced immune responses offer a more broadly applicable alternative. This approach effectively eradicates tumor cells upon light activation while simultaneously initiating durable, systemic immune effects.^[3]^ The integration of phototherapy with immunotherapy not only enhances tumor immunogenicity but also improves therapeutic efficacy across diverse patient populations.

**Scheme 1:**
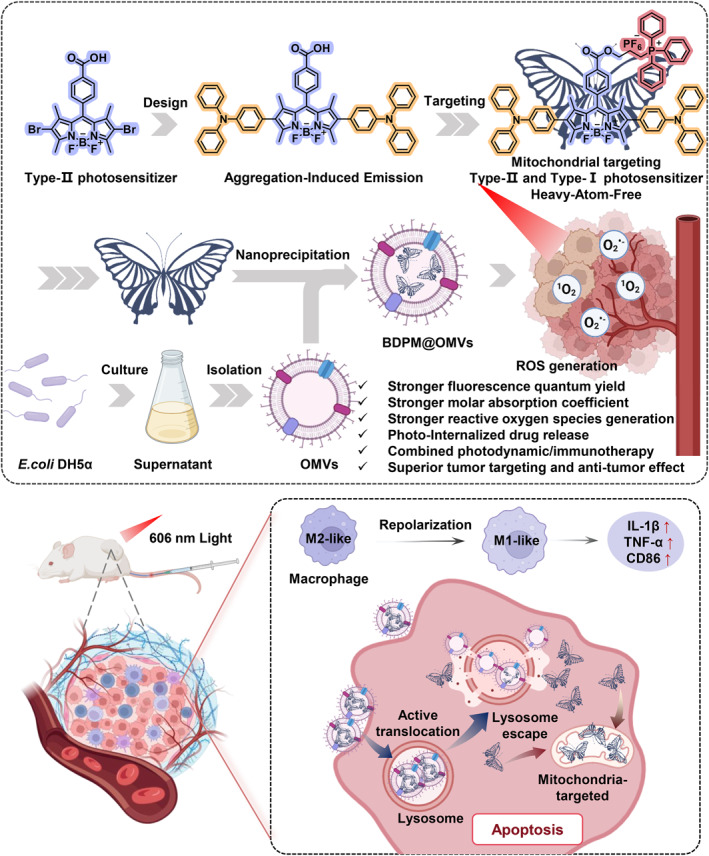
Schematic of the preparation of bacterial outer vesicle‐loaded heavy‐atom‐free photosensitizers for combined photodynamic and immunotherapy.

Bacterial outer membrane vesicles (OMVs) are bilayered nanostructures secreted by Gram‐negative bacteria, and they have recently emerged as highly promising therapeutic delivery carriers. Typically ranging from 20 to 200 nm,[^4^, ^5^, ^6^] OMVs are composed of lipopolysaccharides (LPS), peptidoglycans, periplasmic proteins, and other outer membrane components rich in pathogen‐associated molecular patterns.[^7^, ^8^] A growing body of evidence shows that OMVs can effectively recruit immune cells, initiate antitumor immune responses, and enhance immune‐mediated cytotoxicity by reprogramming macrophages toward the pro‐inflammatory M1 phenotype.[^9^, ^10^, ^11^] Similar to other extracellular vesicles, OMVs exhibit excellent biocompatibility and can be engineered for multifunctional applications. Notably, as key constituents of the tumor microenvironment, bacteria tend to colonize hypoxic regions, which endows OMVs with inherent tumor‐targeting capabilities via hypoxia‐directed chemotaxis.[^12^, ^13^, ^14^] This unique property allows OMVs to retain their intrinsic biological functions while serving as versatile delivery vehicles for therapeutic agents, including small‐molecule drugs, antibodies, and RNA.[^15^, ^16^, ^17^] Therefore, OMVs offer an attractive strategy for enhancing therapeutic outcomes by providing efficient targeting, penetration, and accumulation in hypoxic solid tumors.

Photodynamic therapy (PDT) is a cutting‐edge treatment that combines drugs with a specific light source.[^18^, ^19^, ^20^] Its mechanism relies on photosensitizers that undergo photochemical reactions upon irradiation, converting oxygen into highly cytotoxic reactive oxygen species,^[21]^ such as singlet oxygen (^1^O_2_),^[22]^ superoxide anions (O_2_^•−^),^[23]^ and hydroxyl radicals (•OH).^[24]^ Due to its minimal invasiveness, reduced side effects, and synergistic potential, PDT is widely recommended for treating solid tumors like melanoma and ductal carcinoma.[^20^, ^25^] However, the clinical success of PDT is highly dependent on the photosensitizers used. Conventional photosensitizers often suffer from poor water solubility, leading to aggregation in physiological conditions and a subsequent decline in ROS production efficiency due to aggregation‐caused quenching (ACQ).[^26^, ^27^] To address this, Tang et al. introduced a novel class of photosensitizers that exhibit an aggregation‐induced emission (AIE) effect, where aggregation enhances fluorescence and ROS generation, significantly improving tumor‐killing efficacy.^[28]^ Despite these advancements, a major clinical limitation of PDT is the phototoxicity to normal tissues, a result of inadequate tumor targeting by small organic molecules and their “always‐on” effect.[^29^, ^30^] Thus, developing new photodynamic agents with efficient tumor targeting and minimized off‐target effects is critical for overcoming these challenges. Furthermore, because cancer progression involves multiple signaling pathways, a single treatment often fails to achieve optimal therapeutic outcomes.[^31^, ^32^, ^33^] In contrast, combining multiple targets or mechanisms can yield a synergistic effect that surpasses the sum of individual treatments while effectively counteracting tumor drug resistance.[^34^, ^35^, ^36^, ^37^]

In recent years, OMV‐based photosensitizer systems have emerged as promising platforms for photodynamic immunotherapy by leveraging the intrinsic immunostimulatory properties of bacterial vesicles and their capacity for drug delivery. However, most reported OMV‐based PDT systems primarily employ OMVs as passive carriers to enhance tumor accumulation or immune activation, while the intracellular fate of photosensitizers and the spatiotemporal control of photodynamic damage remain largely unexplored.[^38^, ^39^] Moreover, many of these systems rely on conventional, heavy‐atom‐containing photosensitizers, which often suffer from ACQ, dark toxicity, and compromised performance under hypoxic tumor conditions. Herein, we report a mechanistically distinct OMV‐based photodynamic immunotherapy platform, BDPM@OMVs, which integrates photosensitizer molecular engineering, stepwise subcellular targeting, and immune microenvironment reprogramming into a single system. A novel heavy‐atom‐free AIE photosensitizer (BDPM) was rationally designed to enable efficient Type I/Type II dual‐mode ROS generation and intrinsic mitochondrial targeting. Upon OMV‐mediated cellular internalization, BDPM@OMVs undergo light‐triggered photochemical internalization, inducing lysosomal disruption and subsequent mitochondrial translocation of BDPM, thereby achieving sequential lysosome‐to‐mitochondria photodynamic amplification. Importantly, beyond serving as delivery vehicles, OMVs actively participate in antitumor immunity by promoting macrophage repolarization from the M2 to the M1 phenotype. This unique combination endows BDPM@OMVs with robust photodynamic efficacy under hypoxic conditions and synergistic photodynamic–immunotherapeutic activity against solid tumors. Collectively, this work (Scheme 1) establishes a new design paradigm for OMV‐based photosensitizer systems by emphasizing subcellular photodynamic precision and immune modulation, rather than carrier function alone.

## 2 RESULT AND DISCUSSION

### 2.1 Design and performance of photosensitizer

The introduction of heavy atoms is commonly used to enhance the efficiency of ROS generation by photosensitizers. However, this approach often leads to increased dark toxicity and reduced photostability, which can be detrimental to their biological applications.^[40]^ Enhancing the intramolecular charge transfer (ICT) effect is a promising strategy to lower the highest occupied molecular orbital lowest unoccupied molecular orbital energy gap, thereby increasing the likelihood of intersystem crossing and improving ROS generation efficiency.^[41]^ Fluoroborodipyrrole, a strong electron‐withdrawing group, can significantly enhance the ICT effect. In this work, we developed **BDPM**, a novel heavy‐atom‐free BODIPY photosensitizer featuring an AIE effect. The triphenylamine groups resemble the wings of a butterfly, with the fluoroboron bipyrrole acting as the body and triphenylphosphine as the antenna guiding the molecule toward mitochondria. The butterfly‐shaped molecule, due to the propeller‐like configuration of the two triphenylamine groups, significantly suppresses the formation of intramolecular π‐π stacking, facilitating the enhancement of fluorescence brightness. **BDPM** was synthesized by using two molecules of triphenylamine as the electron donor and fluoroborodipyrrole as the electron acceptor.

**FIGURE 1:**
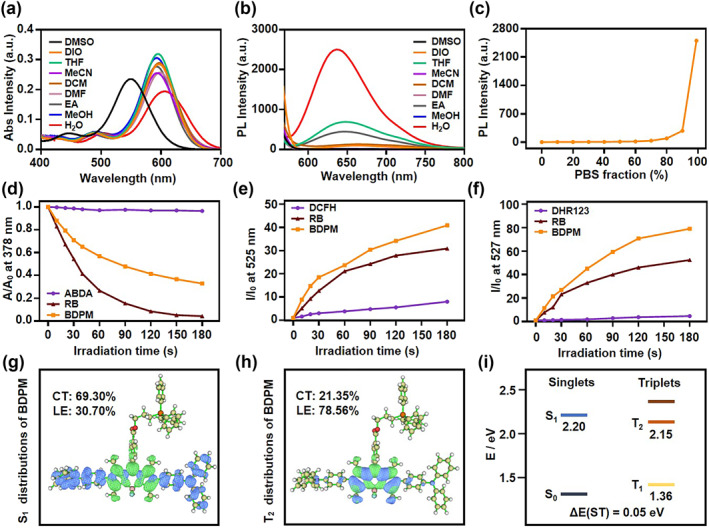
Evaluation of optical properties and reactive oxygen species generation properties of **BDPM**. (a) UV‐Vis absorption spectra of **BDPM** in different solvents. (b) Fluorescence emission spectra of **BDPM** in different solvents. (c) Plot of fluorescence intensity versus in the mixture of DMSO/PBS with different PBS fractions fw. (d) Relative absorption intensity (A/A_0_) of ABDA at 378 nm, (e) relative PL intensity (I/I_0_) of DCFH at 525 nm, and (f) relative PL intensity (I/I_0_) of DHR123 at 527 nm as a function of irradiation time in the presence of **BDPM** or RB in PBS. (g and h) S1 and T2 distributions of **BDPM**. (i) Calculated energy levels of **BDPM**.

As detailed in the Supporting Information, all compounds were fully characterized using electrospray ionization mass spectrometry (ESI‐MS), ^1^H NMR, and ^13^C NMR (Fig. S21–S29). We systematically characterized the spectroscopic properties of BDPM. As shown in Figure 1a,b, **BDPM** exhibits UV absorption in PBS within the range of 550–650 nm, with a peak absorption at 600 nm. The fluorescence emission of **BDPM** was significantly enhanced by 386‐fold as the water content increased in the DMSO/H_2_O system, demonstrating excellent AIE properties (Figure 1c and Figure S1). The emission spectrum of **BDPM** in the aggregated state spans 600–750 nm, with a maximum at 648 nm, which falls within the near‐infrared region and is beneficial for bioimaging applications due to reduced background fluorescence.

**FIGURE 2:**
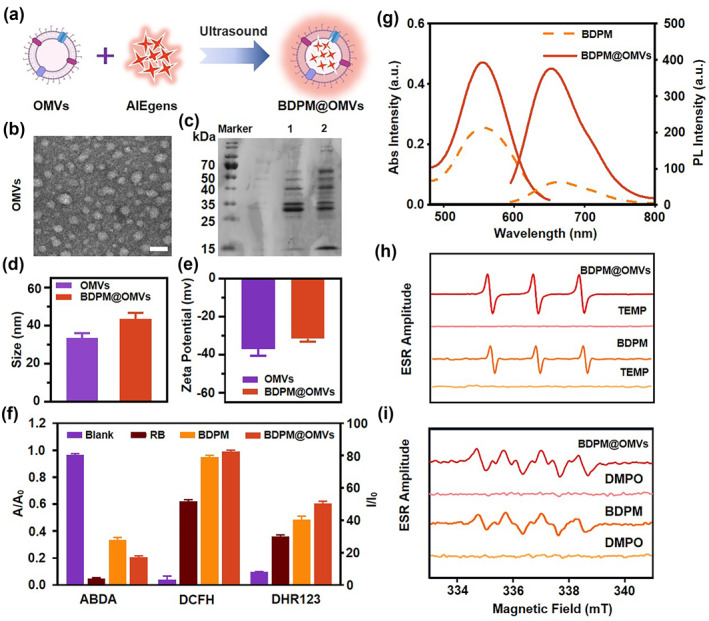
(a) Schematic diagram of ultrasonic preparation of **BDPM@OMV**s. (b) TEM image of OMVs obtained from (E. coli DH5α. scale bar:100 nm. (c) Protein banding analysis of OMVs and **BDPM@OMVs**. (d) Particle size analysis of OMVs and **BDPM@OMVs**. (e) Zeta potential of OMVs and **BDPM@OMVs**. (*n* = 3). (f) Relative absorption intensity (A/A_0_) of ABDA at 378 nm, relative PL intensity (I/I_0_) of DCFH at 523 nm, and relative PL intensity (I/I_0_) of DHR123 at 527 nm as a function of irradiation 180s in the presence of **BDPM** or **BDPM@OMVs** in PBS. (g) UV absorption spectra and fluorescence emission spectra of **BDPM** and **BDPM@OMVs** in PBS. (h) ESR spectra of adducts of TEMP/^1^O_2_ in the presence of PSs with or without irradiation. (i) ESR spectra of adducts of DMPO/O_2_^•−^ in the presence of PSs with or without irradiation. OMVs, outer membrane vesicles; TEM, transmission electron microscopy.

The ^1^O_2_ quantum yield of BDPM was determined using 9,10‐anthracenediyl‐bis(methylene) dimalonic acid (ABDA) in PBS (Figure 1d and Figure S2). Additionally, we assessed the photo‐induced production of ROS and superoxide anions by BDPM using DCFH‐DA (for total ROS) (Figure 1e and Figure S3) and DHR123 (for O_2_^•−^) (Figure 1f and Figure S4). BDPM showed excellent ROS generation ability under white light irradiation, with 42‐fold and 80‐fold increases in fluorescence intensity for DCFH and DHR123, respectively, outperforming the commercial photosensitizer RB. Electron paramagnetic resonance spectroscopy further verified the types of ROS produced by **BDPM**, using 2,2,6,6‐tetramethylpiperidine (TEMP) as a singlet oxygen scavenger and 5,5‐dimethyl‐1‐pyrroline N‐oxide (DMPO) as a superoxide anion scavenger (Figure 2h,i). In conclusion, **BDPM** is a highly efficient type I and type II hybrid photosensitizer.

### 2.2 Theoretical studies on the photodynamic mechanism

To gain a deeper understanding of the photodynamic mechanism, we conducted extensive theoretical computational studies using density functional theory (DFT) and time‐dependent density functional theory calculations to investigate the photophysical and electronic properties of **BDPM**. Initially, we optimized the ground‐state structure using the B3LYP/def2‐SVP functional to achieve the local optimal conformation. Frequency calculations were then performed to confirm the accuracy of the optimized structure. Based on the optimized geometries, we calculated excitation energies using several functionals, including B3LYP/def2‐TZVP, CAM‐B3LYP/def2‐TZVP, PBE0/def2‐TZVP, and M06‐2X/def2‐TZVP, and compared the calculated absorption wavelengths with experimental results. The excitation energies determined by PBE0/def2‐TZVP were found to be closest to the experimental values, enabling us to derive the T‐state excitation energies for this structure.

The results revealed a small energy level difference (Δ*E*(ST) = 0.05 eV) between the S1 and T2 states of **BDPM**, facilitating intersystem crossing (ISC) (Figure 1i). Next, we analyzed the excited‐state electron transitions. The electron‐hole analysis, as shown in Figure 1g,h, demonstrated that the S1 state of **BDPM** exhibited a hybrid CT/local excitation (LE) character upon excitation. In the CT state, CT occurred from the triphenylamine (TPA) moiety to the electron‐accepting group. We further analyzed the ratio of excitation types in the S1 and T2 states using interfragment CT (IFCT).^[42]^ The results (Figure 1g,h, Figure S5) indicated that the transition of **BDPM** from the S1 to the T2 state was accompanied by a significant decrease in the CT character and a notable increase in the LE character (ΔCT% = 47.95), which facilitated the ISC process. These findings suggest that the designed photosensitizer, **BDPM**, is capable of undergoing intersystem crossing, exhibiting significant potential for generating ^1^O_2_.

Among ROS, ^1^O_2_ exhibits higher cytotoxicity, making type II PDT more phototoxic to tumor cells. However, over 90% of solid tumors exhibit hypoxic microenvironments, and the oxygen dependence of type II PDT limits the therapeutic efficacy of corresponding photosensitizers in these tumors. In contrast, type I photoresponses, which involve biocatalytic oxygen recirculation, are less oxygen‐dependent, making type I photosensitizers more effective in hypoxic tumors. Consequently, **BDPM**, a novel hybrid photosensitizer that is free from heavy atoms yet possesses excellent ROS generation capacity, shows significant promise for treating hypoxic tumor cells.

### 2.3 Construction of photosensitizer‐encapsulated OMVs

Outer membrane vesicles are bilayer vesicular spherical nanoparticles with negatively charged surfaces. This lipid bilayer structure not only facilitates the entry of OMVs into host cells but also allows for efficient drug loading within the vesicles.^[43]^ Low‐energy ultrasound was applied to incorporate **BDPM** into the hydrophobic layer of OMVs for effective loading. Initially, OMVs were successfully isolated from E. coli DH5α using ultracentrifugation, achieving a concentration of 0.72 mg/mL, as determined by the BCA protein quantification assay (Figure S6). The bilayer vesicle structure of the extracted OMVs was confirmed by transmission electron microscopy (Figure 2b). Following this, **BDPM** was efficiently loaded into the OMVs using the ultrasound‐assisted nanoprecipitation method, resulting in the formation of the **BDPM@OMVs** nanocomposite (Figure S7).

To assess whether OMVs were altered before and after the loading of the photosensitizer, we first analyzed the protein profile of OMVs using sodium dodecyl sulfate‐polyacrylamide gel electrophoresis (SDS‐PAGE) (Figure 2c). No significant changes were observed after photosensitizer encapsulation, confirming that the immunogenicity of the OMVs was preserved. The particle sizes observed via. Transmission electron microscopy were consistent with the average sizes determined by dynamic light scattering, which were 33.5 and 43.1 nm, respectively (Figure 2d). The increase in size further supported the successful loading of **BDPM** into the OMVs. Zeta potential measurements (Figure 2e) showed that the potential increased from −37.3 mV to −31.5 mV after **BDPM** loading. These results confirmed that the unique vesicle structure of OMVs was retained, successfully leading to the preparation of the **BDPM@OMVs** hybrid vesicle nanoparticles.

After successfully preparing the OMV hybrids, we investigated the optical and photodynamic properties of **BDPM@OMVs**. The results showed that the UV absorption and fluorescence emission spectra of **BDPM** and **BDPM@OMVs** were consistent (Figure 2g). Notably, **BDPM@OMVs** exhibited higher molar absorption coefficients and fluorescence intensities compared to free **BDPM**, likely due to changes in the aggregation state upon encapsulation. The ROS production efficiency of **BDPM@OMVs** was assessed using three different ROS indicators (Figure 2f). **BDPM@OMVs** generated slightly more ROS than free **BDPM**. This increase may be attributed to the altered aggregation state of **BDPM** within the OMVs. Additionally, **BDPM@OMVs** were capable of producing both singlet oxygen and superoxide anions. Electron paramagnetic resonance spectroscopy further confirmed the types of ROS produced (Figure 2h,i). Notably, **BDPM@OMVs** exhibited higher ROS generation efficiency than RB, regardless of whether **BDPM** was encapsulated in OMVs.

### 2.4 BDPM@OMVs targets cancer cells in vitro

**FIGURE 3:**
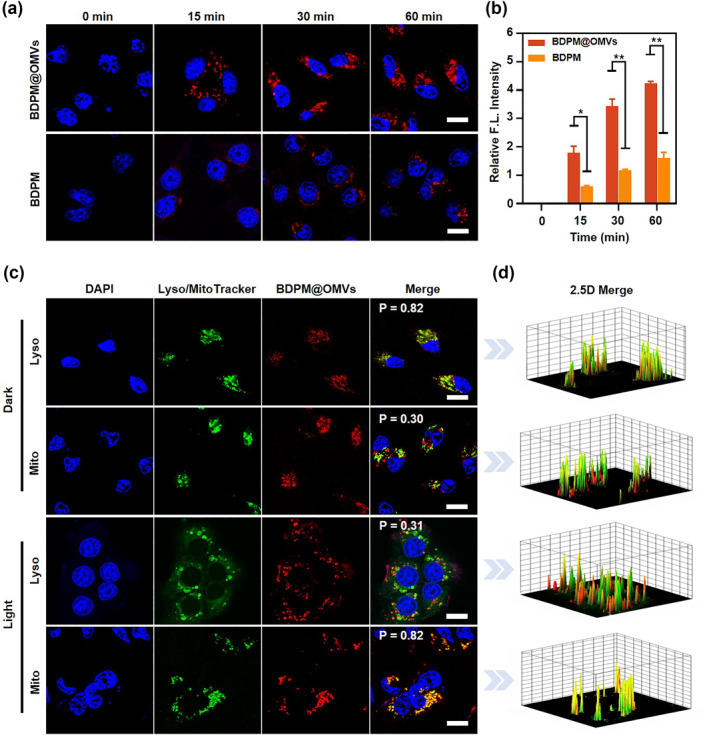
(a) Fluorescence imaging plots and (b) relative fluorescence intensity analysis of **BDPM** (5 μM) and **BDPM@OMVs** (5 μM) incubated with MDA‐MB‐231 cells for different times (0 min, 15 min, 30 min, 60 min). Scale bar: 10 μm. Data are shown as mean ± SD (*n* = 3). *p < 0.05 and **p < 0.01. (c) Co‐localization images and interactive 3D surface Plots of red and green light channels of **BDPM@OMVs** (5 μM) and lysosomal dye LysoTracker‐Green co‐incubated/mitochondrial dye MitoTracker‐Green before and after light exposure. Scale bar: 10 μm. Red channel: excitation at 565 nm, emission collected at 630–700 nm. Green channel: excitation at 488 nm, emission collected at 510–550 nm. Blue channel: excitation at 408 nm, emission collected at 460–500 nm.

Due to the bilayer vesicle structure of OMVs, they can easily enter tumor cells through both membrane fusion and endocytosis.^[44]^ The persistent fluorescence of **BDPM** allowed it to track the internalization of OMVs by tumor cells. To confirm the ability of **BDPM@OMVs** to be internalized by tumor cells in vitro, cellular uptake experiments were conducted. As shown in Figure 3a,b, **BDPM@OMVs** demonstrated more rapid and efficient internalization into tumor cells compared to free **BDPM**. Additionally, the positive charge of **BDPM** enables it to specifically stain mitochondria in living cells after being released inside the cell. As nano‐sized carriers, OMVs are transported into lysosomes after cellular uptake. Upon generating sufficient ROS through light activation, the permeability of the lysosomal membrane is altered, releasing both **BDPM** and OMVs in a process known as photochemical internalization.

To verify this cellular uptake and internalization process, organelle co‐localization experiments were conducted in living cells. The results (Figure 3c,d, and Figure S8) showed that MDA‐MB‐231 cells incubated with **BDPM@OMVs** for 30 min exhibited high co‐localization of **BDPM's** red fluorescence with the green fluorescence of lysosomes (*p* = 0.82), indicating that **BDPM@OMVs** were primarily localized in lysosomes. After 620 nm laser irradiation for 3 min, both green and red fluorescence shifted from an aggregated to a diffuse state, and the cells appeared to bulge, with the co‐localization coefficient decreasing (*p* = 0.31). Meanwhile, the co‐localization coefficient of mitochondrial green fluorescence and **BDPM's** red fluorescence increased from 0.30 to 0.82 before and after irradiation. These results suggest that **BDPM@OMVs** first enter lysosomes following cellular uptake, and after light activation, **BDPM** escapes from lysosomes and localizes in mitochondria. **BDPM** exhibited excellent mitochondrial targeting ability (Figure S9). This innovative design not only facilitates multiple organelle damage but also prevents OMVs from being digested and degraded by cellular enzymes after entering the lysosome, thereby synergistically enhancing the efficacy of photodynamic and immunotherapy.

### 2.5 Eradication of cancer cells in vitro

**FIGURE 4:**
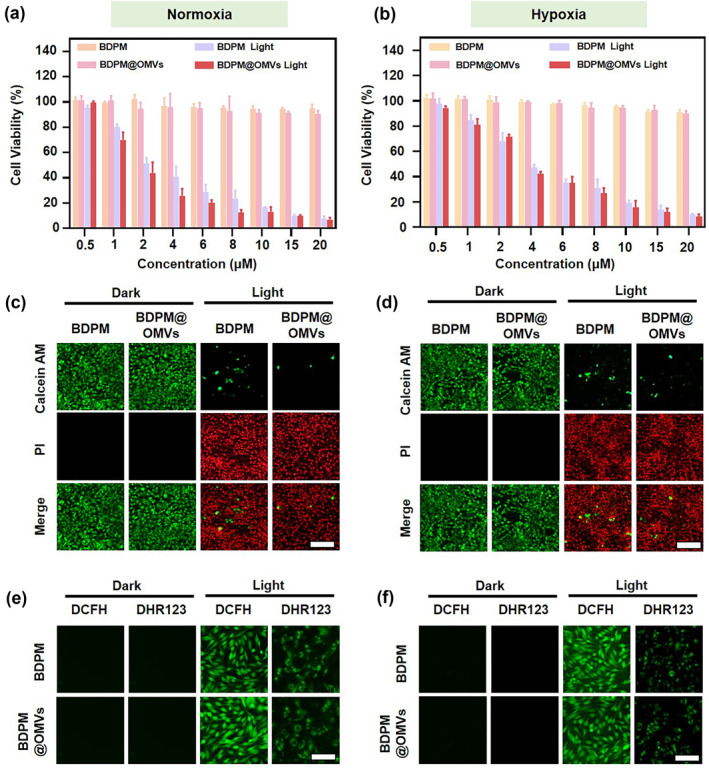
Relative tumor cell viability following PBS, **BDPM** (5 μM), or **BDPM@OMVs** (5 μM) treatment and subsequent white light under normoxia (a) or hypoxia (b) conditions, as assessed by an MTT assay. All experiments are performed in triplicates (*n* = 3), and data are presented as mean ± SD. Fluorescent images of MDA‐MB‐231 cells after various treatments under normoxia (c) or hypoxia (d) conditions. The cells are stained with Calcein‐AM for live cells and propidium iodide (PI) for dead cells. Scale bar: 100 μm. Fluorescent images of photoinduced (1 W/cm^2^, 15 min) ROS in MDA‐MB‐231 cells after incubation with **BDPM** and **BDPM@OMVs** under normoxia (e) and hypoxia (f). DCFH and DHR123 were used as fluorescence probes. Scale bar: 20 μm.

The notable photodynamic activity of **BDPM@OMVs** prompted us to explore whether cancer cells could be effectively inactivated by the ROS generated by **BDPM@OMVs** during co‐incubation. Triple‐negative breast cancer, a “cold” tumor with a poor response to conventional immunotherapy, is the most aggressive and dangerous type of breast cancer. Therefore, **BDPM** or **BDPM@OMVs** were co‐cultured with the triple‐negative breast cancer cell line MDA‐MB‐231 under both normoxia and hypoxia conditions, followed by irradiation with white light for 30 min. Cell viability was assessed using MTT analysis. The results (Figure 4a,b) indicated that neither **BDPM** nor **BDPM@OMVs** were significantly toxic to cells under dark conditions. However, after white light irradiation, cell viability was reduced by 80% when the concentration of **BDPM@OMVs** exceeded 6 μM. Even under anoxic conditions, both **BDPM** and **BDPM@OMVs** demonstrated the ability to eradicate MDA‐MB‐231 cells, likely due to the low oxygen dependence of type I photodynamic therapy. Additionally, **BDPM@OMVs** also showed high photoinactivation efficiency in eliminating the murine‐derived breast cancer cell line 4T1 (Figure S10).

The phototoxicity of **BDPM@OMVs** toward MDA‐MB‐231 cells was further evaluated using fluorescence imaging. **BDPM@OMVs** were co‐cultured with MDA‐MB‐231 cells for 30 min and then exposed to white light irradiation for 30 min. Subsequently, the cultured cells were stained using the LIVE/DEAD™ Cell Imaging Kit. Consistent with the MTT results (Figure 4c and Figure S11), most of the MDA‐MB‐231 cells were stained with propidium iodide (PI), indicating that the cell membranes were disrupted and the cells had undergone cell death. In contrast, under dark conditions, MDA‐MB‐231 cells were predominantly stained with Calcein‐AM (CAM), indicating the low dark toxicity of **BDPM@OMVs**. Even under hypoxic conditions (Figure 4d), the same concentration of **BDPM@OMVs** eradicated most of the cancer cells, with only a few surviving. These results demonstrate that **BDPM@OMVs** are not only effectively taken up by cancer cells but also efficiently eradicated under light‐activated conditions.

To further validate that **BDPM@OMVs** can be photoactivated to produce ROS even after uptake by cancer cells, we examined the type and levels of ROS in MDA‐MB‐231 cells in the presence of **BDPM@OMVs** using various optical probes. The total intracellular ROS content was assessed using DCFH‐DA, and the results (Figure 4e,f) indicated that **BDPM@OMVs** were effectively photoactivated to generate substantial amounts of ROS in MDA‐MB‐231 cells under both normoxia and hypoxia conditions. The green fluorescence signal was diminished or even disappeared after the addition of sodium azide, a quencher of singlet oxygen (Figure S12a).

Subsequently, intracellular superoxide anion production was investigated using DHR123, and fluorescence imaging revealed (Figure 4e,f) that **BDPM@OMVs** significantly increased intracellular mitochondrial superoxide anion levels under both normoxia and hypoxia conditions. Fluorescence was also quenched (Figure S12b) by the addition of the superoxide anion quencher L‐ascorbic acid (Vc). These results further confirm that **BDPM@OMVs** can be photoactivated to produce both singlet oxygen and superoxide anions. Even under anoxic conditions, **BDPM@OMVs** can enhance intracellular ROS levels through photochemical processes, leading to the effective eradication of cancer cells.

### 2.6 Apoptosis induced by BDPM@OMVs

**FIGURE 5:**
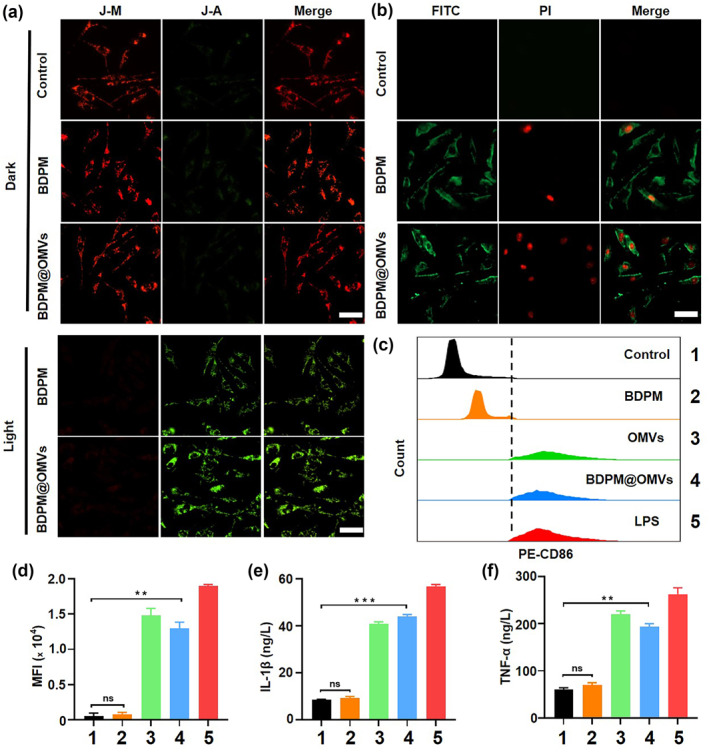
(a) Fluorescence imaging of mitochondrial membrane potential (MMP) in MDA‐MB‐231 cells incubated with **BDPM** and **BDPM@OMVs** (darkness or white light) via subsequent JC‐1 dye assay. Scale bar = 20 μm. (b) Death of MDA‐MB‐231 cells induced by **BDPM** and **BDPM@OMVs** with irradiation and staining with dual fluorescence of annexin V‐FITC/propidium iodide (PI). Scale bar = 20 μm. (c) PE‐CD86 flow‐through results and (d) fluorescence quantification plots of IL‐4 pretreated RAW264.7 cells after treatment with different groups. (e) IL‐1β and (f) TNF‐α assay of IL‐4 pretreated RAW264.7 cells after treatment with different groups. Data are shown as mean ± SD (*n* = 3). **p < 0.01 and ***p < 0.001.

The experimental data above suggest that **BDPM@OMVs** is an effective PDT agent for tumor cell eradication. To further elucidate the detailed therapeutic mechanism of **BDPM@OMVs**, we assessed the death pattern of MDA‐MB‐231 cells. Apoptosis is the most common form of cell death induced by PDT. Once mitochondria are damaged, they lose membrane potential, which triggers apoptosis. Given the mitochondrial targeting of **BDPM**, the alteration in mitochondrial membrane potential (MMP) was detected using JC‐1 (Figure 5a). MDA‐MB‐231 cells in the control and dark groups exhibited intense red fluorescence, indicating high MMP values. In contrast, both **BDPM** and **BDPM@OMVs** led to a decrease in MMP following white light irradiation, as evidenced by enhanced green fluorescence, which reflects mitochondrial dysfunction and correlates with the cytotoxicity assay results.

Additionally, we examined apoptosis using dual fluorescence from membrane‐conjugated protein V‐FITC and PI, where the green fluorescence of FITC indicates apoptosis, and the red fluorescence of PI marks cell death. As shown in Figure 5b, no obvious red or green fluorescence was observed in the control and dark groups. In contrast, **BDPM** and **BDPM@OMVs‐**treated cells exhibited enhanced green and red fluorescence after irradiation. Some apoptotic cells were only labeled with FITC, indicating that they were undergoing apoptosis.

OMVs are potent immunomodulators, and thus, OMVs released through photointernalization are expected to interact with macrophages and exert an immunomodulatory effect. This was investigated by examining the repolarization of macrophages. In the tumor microenvironment, M2‐type macrophages predominate and secrete anti‐inflammatory factors such as IL‐10 and TGF‐β, which dampen the immune response. In contrast, M1‐type macrophages actively promote the immune response by secreting TNF‐α and IL‐1β. Morphologically, M2‐type macrophages typically exhibit an oval, omelet‐like shape, whereas M1‐type macrophages display a mixture of omelet‐like and spindle‐shaped forms. As shown in Fig. S13, we successfully induced RAW264.7 macrophages to polarize into the M2 type using IL‐4, resulting in an omelet‐like shape. Following co‐incubation with lipopolysaccharide (LPS) (an M1‐type macrophage inducer) and **BDPM@OMVs**, the M2‐type macrophages exhibited altered morphology, with some cells developing pseudopodia. These results suggest that LPS present in **BDPM@OMVs** may effectively promote the transition of M2 macrophages to the M1 phenotype.

To further validate the production of M1 macrophages, extracellular cytokines TNF‐α and IL‐1β were measured using ELISA kits. The results indicated that single‐molecule **BDPM** did not stimulate the polarization of RAW264.7 cells. In contrast, both OMVs and **BDPM@OMVs**, similar to LPS, effectively promoted polarization and upregulated the secretion of pro‐inflammatory cytokines TNF‐α and IL‐1β in M1 macrophages (Figure 5e,f). To corroborate these findings, the expression of CD86, a marker commonly expressed in M1 macrophages, was analyzed by flow cytometry. The flow cytometry results (Figure 5c,d) showed significant PE fluorescence signals in both the OMVs and **BDPM@OMVs** groups. Together, these experimental results suggest that OMVs have the potential to promote the repolarization of M2 macrophages to M1 macrophages, thereby enhancing immunotherapy by modulating the immunosuppressive tumor microenvironment.

### 2.7 Anti‐tumor efficacy of photodynamic immunotherapy in vivo

**FIGURE 6:**
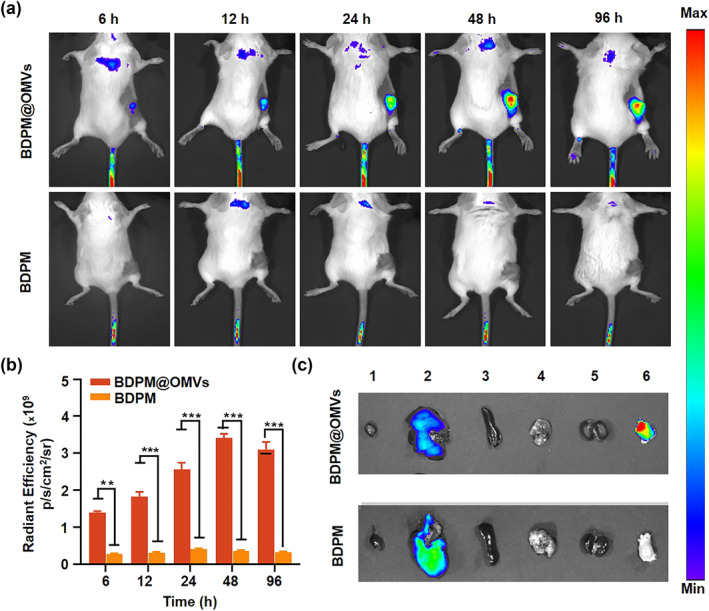
(a) Imaging of mouse tumors following intravenous injection of **BDPM** or **BDPM@OMVs** (100 μL, 100 μM) at various time points. (b) Relative fluorescence intensity analysis of tumor sites after tail vein injection of the same concentration of **BDPM** and **BDPM@OMVs**. Data are shown as mean ± SD (*n* = 3). *p < 0.05 and **p < 0.01. (c) Fluorescence imaging of excised main organs and tumors at 48 h post‐injection. (1, heart; 2, liver; 3, spleen; 4, lungs; 5, kidneys; 6, tumors).

The targeting and therapeutic effects of OMVs on tumors were further evaluated using a 4T1 mouse breast cancer model. A subcutaneous breast cancer tumor model was established by inoculating BALB/c mice with the 4T1 mouse breast cancer cell line. Following tail vein injection of **BDPM@OMVs**, in vivo fluorescence distribution was assessed using an IVIS imaging system with excitation/emission wavelengths of 550/660 nm (Figure 6a,b, and Figure S14). Initially, no fluorescence signal was observed at the tumor site. However, after 6 h, fluorescence signals appeared at the tumor site, indicating the accumulation of **BDPM@OMVs**. Over time, the fluorescence signal at the tumor site gradually increased, peaking at 48 h. Ex vivo tissue imaging of major organs and tumors at 48 and 96 h revealed strong fluorescence from the photosensitizer in the liver and tumor sites at 48 h (Figure 6c and Figure S15). By 96 h, fluorescence in both the liver and tumor sites had significantly decreased. In contrast, mice injected with **BDPM** showed no significant fluorescence at the tumor site. These results demonstrate that **BDPM@OMVs** effectively target tumor tissues, owing to the efficient tumor‐targeting capability of OMVs, and are metabolized from major organs after 48 h, minimizing toxicity to normal tissues.

**FIGURE 7:**
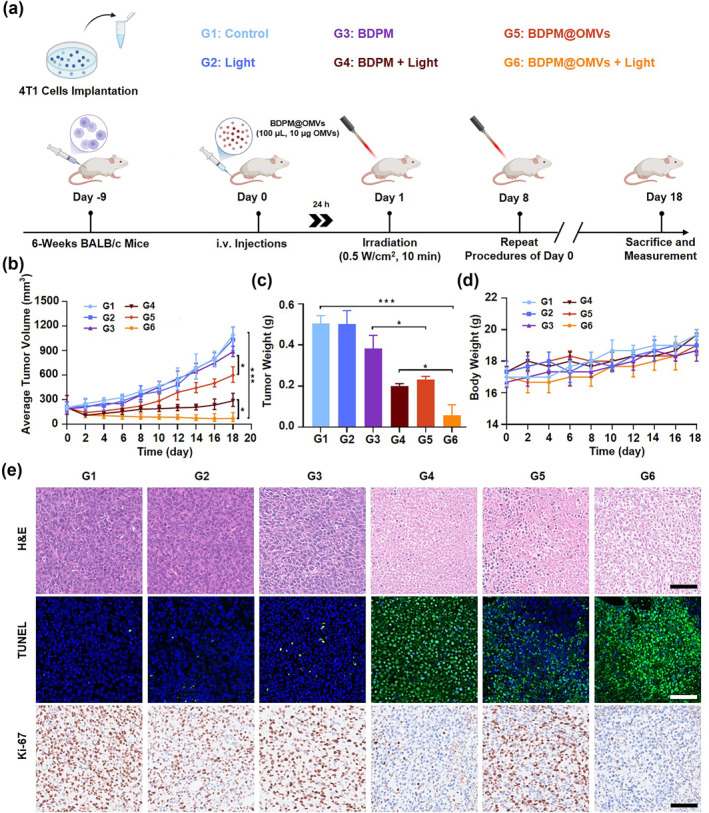
(a) Timeline of photodynamic‐immunotherapy in a 4T1 hormonal mouse model. (b) Mean tumor volume of the 4T1 hormonal mouse model over an 18‐day treatment cycle. (c) Tumor weight of 4T1 hormonal mouse model at 18 days. (d) Mean body weight of the 4T1 hormonal mouse model at 18‐day treatment cycle. Data are shown as mean ± SD (*n* = 3). *p < 0.05 and **p < 0.01. (e) H&E, TUNEL and Ki67 staining of tumor tissues from different groups of 4T1 hormonal mice. Scale bar = 100 μm.

Based on the data demonstrating the effective tumor tissue targeting and therapeutic efficacy of **BDPM@OMVs**, we further investigated their anti‐tumor efficacy in 4T1 tumor‐bearing mice. Specifically, 4T1 tumor‐bearing mice were divided into six groups (*n* = 3). Following tail vein injection, the mice were irradiated with or without 606 nm light (1 W/cm^2^, 10 min) after 12 h. Treatment was repeated once, with light irradiation at 7‐day intervals, and all mice were euthanized for tumor collection after 18 days (Figure 7a). The results (Figure 7b,c and Figure S16) showed that only the **BDPM@OMVs** with light irradiation group exhibited approximately 85% inhibition of tumor growth. The OMVs‐only group showed a modest inhibitory effect similar to that of the **BDPM**‐only light irradiation group. Throughout the treatment period, the body weight of mice in all experimental groups remained stable (Figure 7d). These findings indicate that **BDPM@OMVs** offer superior biocompatibility and effective anti‐tumor activity, further highlighting the benefits of combining PDT with immunotherapy.

To further evaluate the anti‐tumor efficacy of **BDPM@OMVs**, we conducted a series of pathological analyses, including H&E, TUNEL, and Ki67 staining (Figure 7e) on tumors isolated from the tumor‐bearing mice. H&E staining revealed that in the **BDPM** + Light, **BDPM@OMVs**, and **BDPM@OMVs** + Light groups, only a few cells remained morphologically intact, with many showing shrunken or ruptured nuclei. TUNEL staining demonstrated a significant increase in apoptotic cells, indicated by green fluorescence. Additionally, Ki67 staining showed a reduction in the proportion of proliferating tumor cells, as evidenced by the decreased light brown staining. Among these, the **BDPM@OMVs** + Light group exhibited the most pronounced effects on cell death and tumor suppression. These results confirm that **BDPM@OMVs**‐induced photoimmunotherapy can achieve excellent anti‐tumor efficacy.

To assess the biocompatibility of **BDPM@OMVs**, erythrocyte lysis experiments were conducted. Using PBS as a negative control and deionized water as a positive control, no significant hemolysis (greater than 5%) was observed, even at a concentration of **BDPM@OMVs** up to 200 μg/mL. Furthermore, H&E staining of major organs (heart, liver, spleen, lungs, and kidneys) from 4T1 tumor‐bearing mice revealed no apparent pathological changes (Figure S17). Serum biochemistry analysis, including measurements of alanine aminotransferase (ALT), aspartate aminotransferase (AST), creatinine (CREA), and blood urea nitrogen, showed values within the normal range, indicating no significant hepatorenal toxicity (Figure S18). Finally, the MTT assay on L‐02 normal human hepatocytes and RAW264.7 macrophages showed cell viability above 80% across the tested concentration range (Figure S19). These findings demonstrate that **BDPM@OMVs** exhibit excellent biological safety.

## 3 CONCLUSION

In this study, we developed **BDPM@OMVs**, a novel nanoplatform combining a heavy‐atom‐free AIE photosensitizer (**BDPM**) with bacterial OMVs. This platform is the first OMV‐based system designed for photodynamic immunotherapy. Our **BDPM** photosensitizer exhibits both Type‐I and Type‐II photodynamic activities, enabling efficient ROS generation without the need for heavy atoms, thus outperforming conventional photosensitizers like Rose Bengal. **BDPM@OMVs** leverage the dual benefits of PDT and immunomodulation. The OMV carriers facilitate targeted delivery to tumor sites and enable the controlled release of **BDPM** within tumor cells, leading to precise tumor‐specific ROS generation and apoptotic cell death. Simultaneously, the OMVs enhance antitumor immunity by promoting macrophage repolarization. This synergistic approach significantly improves therapeutic efficacy while reducing off‐target toxicity. In conclusion, **BDPM@OMVs** represent a transformative strategy for cancer treatment. By integrating targeted photodynamic therapy with immune modulation, this platform selectively eradicates tumors and opens new avenues for advancing the field of photodynamic immunotherapy.

## AUTHOR CONTRIBUTIONS

The manuscript was drafted and reviewed through the contributions of all authors. All authors have given approval to the final version of the manuscript.

## CONFLICT OF INTEREST STATEMENT

The authors declare no conflicts of interest.

## ETHICS STATEMENT

All the animal experiments involved in this work were approved by the animal ethics committee of Central South University (Changsha, China) (license no. XMXH–2023‐1384).

## Supporting information

Supporting Information S1

## Data Availability

Data will be made available on request.
