# Supplementary material for: Stepwise mitochondria‐targeted photodynamic immunotherapy enabled by an outer membrane vesicles‐based nanoplatform for synergistic tumor ablation and immune reprogramming
Source: Smart Mol. 2026 Feb 19;4(2):e70039. doi: 10.1002/smo2.70039 (PMC13317570; doi:10.1002/smo2.70039)
Supplement: Supplementary file 1 — Supporting Information S1 [file SMO2-4-e70039-s001.docx]

Supporting Information

**Stepwise Mitochondria-Targeted Photodynamic Immunotherapy Enabled by an OMV-Based Nanoplatform for Synergistic Tumor Ablation and Immune Reprogramming**

Xiang Cheng, Ziheng Luo, Ying Yin, Duoyang Fan, Ruyan Xie, Yanpeng Fang, Xiaohui Liu, Dayan Xiong, Wei Liu, Seraphine V. Wegner, Fei Chen,* Wenbin Zeng,*

**1. Materials and Experiments**

*1.1 Materials and Chemicals.*

All chemicals used in the synthesis were obtained from commercial suppliers and were used without further purification. The raw materials required for the photosensitizer synthesis were sourced from Energy Chemicals. 2,2,6,6-Tetramethyl-4-piperidone (TEMP), 5,5-dimethyl-1-pyrroline-N-oxide (DMPO), 9,10-anthracenyl-bis(methylene)dipropanedioic acid, 2',7'-dichlorofluorescein diethyl ester, and dihydro Rhodamine 123 were purchased from Sigma-Aldrich. The BCA protein concentration assay kit, pyridinium iodide, Calcein AM, Lyso-Tracker Green, Mito-Tracker Green, Hoechst 33342, and JC-1 were acquired from Beyotime. The PE-anti-mouse-CD86 antibody was purchased from Pike Biologicals, while Interleukin 4 was sourced from Nearshore Protein. ELISA kits were procured from Jingmei Biotechnology Co.

*1.2 General instruments*

NMR spectra were recorded on a Bruker Advance Wave spectrometer with tetramethylsilane (TMS) as the internal standard. High-resolution mass spectrometry (HRMS) was conducted using an Orbitrap Velos Pro LC-MS spectrometer (Thermo Scientific, USA). Nanoparticle morphology was characterized using a Tecnai G2 Spirit Twin transmission electron microscope (Thermo Scientific, USA). UV-visible spectra were acquired with a Shimadzu UV-2450 UV-visible spectrophotometer. PL spectra were collected on a HITACHI-7100 PC Spectro fluorescence photometer (Shimadzu, Japan). MTT assays were performed using a microplate reader (SpectraMax M2, USA). ROS staining, live/dead cell staining, mitochondrial membrane potential staining, and apoptosis staining were visualized using a fluorescence inverted microscope. The diameters of nanoparticles were measured by dynamic light scattering (DLS) on a Nano-ZS ZEN3600 particle sizer (Malvern Instruments). Confocal laser scanning microscopy (CLSM) images were obtained using a TCS SP8 confocal laser scanning microscope (Leica, Germany). Flow cytometry (LSRFortessa, USA) was used for immunoflow data collection. In vivo fluorescence imaging of animals was carried out using the IVIS-Spectrum BL in vivo imaging system.

*1.3* *Synthesis and Characterization*

Synthesis of **M-1**: Triphenylphosphine (1.57 g, 1.20 mmol) was placed in a 25 mL round-bottom flask. To this, 3-bromo-1-propanol (695 mg, 1.00 mmol) and toluene (9 mL) were added dropwise. The reaction mixture was then heated to reflux and maintained for 24 hours. After completion, the solvent was removed under reduced pressure using a rotary evaporator. The resulting residue was washed with ethyl ether, filtered, and dried to yield a white solid (1.66 g, 86% yield). ^1^H NMR (600 MHz, DMSO-*d6*) δ ppm 7.91 (dd, *J* = 10.4, 4.4 Hz, 3H), 7.83 (dd, *J* = 12.5, 7.3 Hz, 6H), 7.78 (td, *J* = 7.8, 3.6 Hz, 6H), 4.88 (s, 1H), 3.58 (ddd, *J* = 30.5, 18.0, 5.8 Hz, 4H), 1.67 (s, 2H). ^13^C NMR (151 MHz, DMSO-d6) δ ppm 135.38, 134.10, 130.76, 119.31, 118.74, 60.75, 25.83. HRMS (m/z): calculated for C21H22BrOP [M-Br]^+^: 321.1403; found: 321.1405.

Synthesis of **1**: P-formylbenzaldehyde (750 mg, 5.00 mmol) and 2,4-dimethylpyrrole (1.045 g, 11.00 mmol) were placed in a 500 mL round-bottom flask, to which 100 mL of tetrahydrofuran (THF) was added under a nitrogen atmosphere. After stirring the mixture at room temperature, protected from light, for 15 minutes, a few drops of trifluoroacetic acid were added, resulting in a red solution. The reaction was continued for 12 hours, with stirring and protection from light. Then, 2,3-dichloro-5,6-dicyano-p-benzoquinone (1.135 g, 5.00 mmol) and an additional 100 mL of THF were added, and the mixture was stirred for 4 hours. The round-bottom flask was then cooled in an ice bath, and triethylamine (20 mL) was added dropwise over 15 minutes. After removal from the ice bath and allowing the mixture to return to room temperature, boron trifluoride ethyl ether complex (20 mL) was added dropwise and the reaction was allowed to proceed overnight at room temperature. The reaction progress was monitored by thin-layer chromatography (DCM : MeOH = 50 : 1, v/v). The solvent was removed under reduced pressure using a rotary evaporator. The residue was extracted with water (H_2_O) and dichloromethane (DCM), and the organic layer was washed with saturated saline. The lower layer was separated, dried over anhydrous magnesium sulfate (MgSO_4_), and evaporated under reduced pressure to remove the solvent, yielding a dark green solid mixture. The mixture was dissolved in an appropriate amount of DCM, and 100–200 mesh silica gel powder was added to the sample. The sample was then purified by column chromatography using 200–300 mesh silica gel (DCM : MeOH = 30 : 1, v/v), resulting in the isolation of compound **1** as a red powder (607 mg, 33% yield).

Synthesis of **2**: Compound **1** (500 mg, 1.35 mmol) and N-bromosuccinimide (478 mg, 2.70 mmol) were placed in a 50 mL round-bottom flask. Anhydrous tetrahydrofuran (THF, 15 mL) was added under light-avoidance conditions, and the mixture was stirred overnight at room temperature. The reaction progress was monitored by thin-layer chromatography (DCM : MeOH = 50 : 1, v/v) until completion. Afterward, the solvent was removed under reduced pressure using a rotary evaporator, yielding a red solid mixture. The residue was dissolved in an appropriate amount of DCM and mixed with 100–200 mesh silica gel powder. The sample was then purified by column chromatography using 200–300 mesh silica gel (DCM : MeOH = 80 : 1, v/v), resulting in the isolation and purification of compound **2** as a dark red powder (636 mg, 89% yield).

Synthesis of **3**: Compound **2** (500 mg, 0.95 mmol), **M1** (688 mg, 2.38 mmol), and potassium carbonate (K_2_CO_3_, 393 mg) were placed in a 250 mL three-necked flask, under a nitrogen atmosphere. To this, tetrakis(triphenylphosphine)palladium (20 mg), anhydrous tetrahydrofuran (THF, 45 mL), and water (15 mL) were added. The reaction was carried out at reflux for 24 hours. The progress of the reaction was monitored by thin-layer chromatography (TLC) (DCM : MeOH = 30 : 1, v/v) until complete conversion was observed. Afterward, the solvent was removed under reduced pressure using a rotary evaporator. The residue was extracted with water and dichloromethane (DCM), and the organic layer was washed with saturated saline. The lower layer was dried over anhydrous magnesium sulfate (MgSO_4_), and the solvent was evaporated under reduced pressure to yield a dark purple solid mixture. The mixture was dissolved in an appropriate amount of DCM, and 100–200 mesh silica gel powder was added to the sample. The product was then purified by column chromatography using 200–300 mesh silica gel (DCM : MeOH = 100 : 1, v/v), resulting in the isolation of compound **3** as a purple powder (333 mg, 41% yield).^1^H NMR (500 MHz, CDCl_3_) δ ppm 8.27 (d, *J* =8.1 Hz, 2H), 7.53 (d, *J*=8.1 Hz, 2H), 7.26 (t, *J*=7.8 Hz, 8H), 7.12 (d, *J*=7.8 Hz, 8H), 7.03 (dt, *J*=19.7 Hz, 8.5, 12H), 2.58 (s, 6H), 1.33 (s, 6H). ^13^C NMR (126 MHz, CDCl_3_) δ ppm 170.41, 155.05, 147.60, 146.87, 141.42, 139.86, 138.54, 133.78, 131.03, 130.80, 129.76, 129.30, 128.81, 126.96, 124.61, 123.08, 122.91, 13.08. HRMS (m/z): calculated for C56H45BF2N4O2 [M-H]^+^: 853.3604; found: 853.3519.

Synthesis of **BDPM**: Compound **3** (500 mg, 0.58 mmol), compound **4** (280 mg, 0.70 mmol), 1-(3-dimethylaminopropyl)-3-ethylcarbodiimide hydrochloride (223 mg, 1.16 mmol), and 4-dimethylaminopyridine (3.66 mg, 0.03 mmol) were placed in a 50 mL round-bottom flask and dissolved in anhydrous dichloromethane (DCM, 20 mL). The reaction mixture was refluxed in a water bath under heating conditions. The reaction progress was monitored by thin-layer chromatography (TLC) (DCM : MeOH = 100 : 1, v/v) until completion. Afterward, the solvent was removed under reduced pressure using a rotary evaporator. The residue was extracted with water and DCM, and the organic layer was washed with saturated saline. The lower layer was dried over anhydrous magnesium sulfate (MgSO_4_), and the solvent was evaporated under reduced pressure to yield a dark purple solid mixture. The mixture was dissolved in an appropriate amount of DCM, and 100–200 mesh silica gel powder was added to the sample. The product was then purified by column chromatography using 200–300 mesh silica gel (PE : DCM = 1 : 1, v/v), resulting in the isolation of compound BDPM-Br as a purple powder (440 mg, 65% yield). BDPM-Br was subsequently dissolved in methanol, and a saturated aqueous solution of potassium hexafluorophosphate (KPF_6_) was added. The reaction was stirred at room temperature for 12 hours and further purified by high-performance liquid chromatography (HPLC). ^1^H NMR (500 MHz, DMSO-*d6*) δ ppm 8.19 (d, *J* = 8.2 Hz, 2H), 7.88 (ddd, *J* = 20.1, 10.3, 7.0 Hz, 10H), 7.79 (dd, *J* = 7.6, 3.4 Hz, 5H), 7.68 (d, *J* = 8.2 Hz, 2H), 7.32 (t, *J* = 7.9 Hz, 8H), 7.12 (d, *J* = 8.5 Hz, 4H), 7.06 (t, *J* = 7.2 Hz, 12H), 6.97 (d, *J* = 8.5 Hz, 4H), 4.42 (t, *J* = 5.5 Hz, 2H), 3.89 – 3.78 (m, 2H), 2.47 (s, 6H), 2.07 (d, *J* = 6.4 Hz, 2H), 1.29 (s, 6H). ^13^C NMR (126 MHz, DMSO-*d6*) δ ppm 165.64, 154.56, 147.37, 146.84, 141.18, 139.96, 138.57, 135.48, 134.17, 134.09, 131.34, 130.82, 130.72, 130.12, 129.26, 126.42, 124.98, 123.98, 122.38, 119.12, 118.44, 56.49, 19.02, 13.81, 13.22. HRMS (m/z): calculated for C77H65BBrF2N4O2P [M-PF_6_^-^]^+^: 1157.4901; found: 1157.4941.


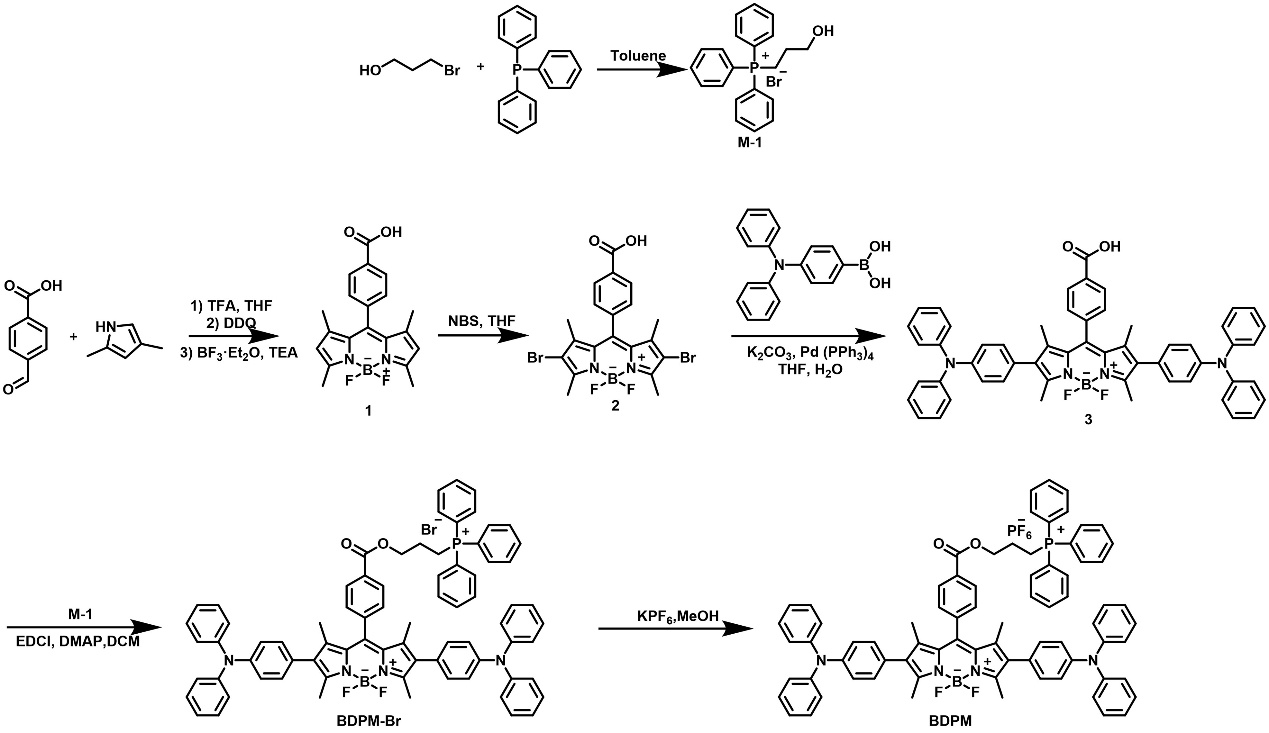


Scheme S1. The synthetic route of the **BDPM**.

*1.4 UV-vis and Fluorescence Spectroscopy*

A total of 2.32 mg of the fluorescent probe **BDPM** compound was weighed and placed in a 1.5 mL EP tube. To this, 1 mL of anhydrous dimethyl sulfoxide was added, and the mixture was shaken to ensure complete dissolution, yielding a **BDPM** masterbatch with a concentration of 2 mM.

Next, 1990 μL of various solvents, including dimethyl sulfoxide (DMSO), 1,4-dioxane (DiO), tetrahydrofuran (THF), acetonitrile (MeCN), dichloromethane (DCM), N,N-dimethylformamide (DMF), ethyl acetate (EA), methanol (MeOH), and deionized water (H_2_O), were prepared. To each solvent, 10 μL of the BDPM masterbatch was added, and the mixture was thoroughly mixed to create **BDPM** solutions with a final concentration of 10 μM in different solvents. The UV absorption and fluorescence emission spectra of the BDPM fluorescent probe in each solvent were then measured.

Subsequently, the 2 mM **BDPM** masterbatch was diluted to a concentration of 10 μM using different volume ratios of mixed H_2_O and DMSO solvents. The DMSO content in the solvent mixtures ranged from 0% to 99% in 10% increments (0%, 10%, 20%, 30%, 40%, 50%, 60%, 70%, 80%, 90%, and 99%). The fluorescence emission spectra of the BDPM probe in these different solvent mixtures were recorded.

*1.5* *Theoretical Calculation*

The initial molecular structure of the compound was generated using the Molecular Operating Environment (MOE). For subsequent analysis, density functional theory (DFT) was employed to perform geometry optimization at the B3LYP/6-311G(d,p) level. Time-dependent DFT (TD-DFT) was used to calculate the excited states at the CAM-B3LYP/6-311G(d,p) level. The Becke–Johnson damping (DFT-D3(BJ)) correction was applied to account for dispersion effects. The solvent effect of water was incorporated using the solvation model based on density (SMD). All DFT calculations were carried out using Gaussian 16. Orbital visualization was achieved with the help of Multiwfn 3.8 and VMD software.

*1.6 Preparation and Characterization of OMVs*

Preparative isolation of OMVs：The Escherichia coli DH5α strain, stored in glycerol at -80 °C, was inoculated into autoclaved LB medium and incubated overnight at 37 °C with shaking at 180 rpm. A small aliquot of the bacterial culture was transferred with a sterilized inoculation loop and streaked onto LB agar plates. The plates were then incubated overnight at 37 °C to obtain isolated monoclonal colonies.

Monoclonal colonies were picked and transferred to fresh LB medium. The bacterial suspension was then mixed with 50% glycerol in a 1:1 (v/v) ratio and stored at -80 °C for future use. This version streamlines the description and improves clarity, making it sound more formal and consistent with scientific writing standards.

Monoclonal colonies of Escherichia coli DH5α were inoculated into LB medium and incubated at 37 °C with shaking at 180 rpm for 6 hours. The culture was then expanded to 2 L by adding fresh LB medium at a 1 : 100 (v/v) ratio. The bacterial concentration was monitored by measuring the optical density at 600 nm (OD600). The cultivation was terminated once the bacteria reached the logarithmic growth phase, as indicated by an OD600 value of 1.0. The culture was subjected to centrifugation at 5000 × g for 30 minutes at 4 °C to remove the majority of the parent bacteria. The supernatant was then filtered through a 0.45 μm sterile needle filter to remove any remaining parent bacteria. The filtered supernatant was concentrated and enriched using an ultrafiltration device with a 100 kDa molecular weight cutoff. The concentrated supernatant was ultracentrifuged at 150,000 × g for 3 hours at 4 °C to isolate the outer membrane vesicles (OMVs). The OMV pellet was resuspended in sterile PBS (pH 7.4) and stored at -80 °C for future experiments. This revision clarifies the steps and improves the structure and flow of the text, making it more concise and aligned with scientific writing conventions.

Protein concentrations of the OMVs were determined using the BCA Protein Assay Kit, following the procedure outlined below: a. Prepare a series of BCA standards with concentrations of 2, 1, 0.5, 0.25, 0.125, 0.0625, and 0 mg/mL using the provided BCA standard solutions and diluents.b. For the microtiter assay, prepare 5.4 mL of BCA working solution, considering the total volume required for all test samples (including 7 BCA standards and 1 sample to be tested). Mix BCA-A and BCA-B in a 50:1 volume ratio. c. Dispense 200 μL of the prepared BCA working solution into each well of a 96-well plate. Use three replicates for each sample (n = 3). Pipette 25 μL of each BCA standard and OMV sample into the corresponding wells. Mix well. Incubate the plate in an incubator at 37 °C for 30 minutes. After incubation, measure the absorbance of each well at 540-590 nm using a microplate reader (enzyme meter) to determine the protein concentration.

*1.7* *Preparation of* ***BDPM@OMVs***

A 10 μM aqueous solution of **BDPM** was prepared, and the **BDPM** solution was added dropwise into the PBS solution of OMVs using a 1 mL syringe under ultrasonic probe-assisted mixing. The solution gradually changed from colorless and transparent to purplish-red as the **BDPM** was incorporated. The mixture was subjected to continued ultrasonication for 40 minutes after the completion of the dropwise addition. After ultrasonication, the solution was filtered through a 0.45 μm sterile needle filter to remove any remaining particulate matter. The filtrate was then placed into a dialysis bag to remove trace amounts of the organic solvent. The resulting solution contained homogeneously dispersed **BDPM@OMVs** nanoparticles. To verify the homogeneity of the **BDPM@OMVs** nanoparticles, a red laser pointer was used to irradiate the nanoparticle dispersion, and the Tyndall effect was observed. Pure water was used as a blank control.

*1.8 Characterization of* ***BDPM@OMVs***

The prepared OMVs and **BDPM@OMVs** were diluted to an appropriate concentration. A 10 μL aliquot of each sample was applied onto a copper grid and allowed to stand at room temperature for 5 minutes. Excess liquid was then removed using filter paper. The samples were negatively stained with 2% (w/v) phosphotungstic acid for 1–2 minutes, followed by washing with pure water three times. The morphology of OMVs and **BDPM@OMVs** was observed under a transmission electron microscope (TEM).

The OMVs and **BDPM@OMVs** were diluted to a suitable concentration and placed in a 1.5 mL cuvette. The particle sizes and zeta potentials of the OMVs and **BDPM@OMVs** were measured using a nanoparticle size and zeta potential analyzer. Each measurement was repeated three times for each sample to ensure reproducibility.

Protein electrophoretic analysis of **BDPM@OMVs**: a. Gel Preparation: Clean the glass plates thoroughly with pure water and assemble them. Add pure water to the gap between the two glass plates and allow it to stand for a while to check for any leaks. Using a pipette, slowly add the prepared gel solution to the glass plates, filling to the desired height. Add a layer of pure water on top to ensure the liquid level is even and to prevent air bubbles. Allow the gel to stand at room temperature until it solidifies. Prepare a 5% concentrated gel solution. After discarding the layer of pure water that separated from the gel, slowly add the concentrated gel solution to the glass plate. Gently insert the comb and allow the gel to solidify at room temperature. b. Sample Preparation: Dilute the prepared OMVs and **BDPM@OMVs** to the desired concentration. Add 5X sample buffer to the samples, mix thoroughly, and then heat the mixture at 95°C for 10 minutes to denature the proteins. c. Electrophoresis: Assemble the glass plates with the prepared gel in the electrophoresis tank. Add an appropriate volume of electrophoresis buffer into the tank. Gently remove the comb and carefully load the protein marker and samples into the wells using a pipette. Once the electrophoresis device is connected, run the electrophoresis at 80 V for 30 minutes, or until the protein marker bands reach the boundary between the concentrated gel and the separating gel. Then, increase the voltage to 120 V and continue electrophoresis for an additional hour. Stop the electrophoresis once the protein marker bands have migrated to the desired position. d. Coomassie Brilliant Blue Staining and Decolorization: After electrophoresis, carefully remove the concentrated gel and wash the separating gel with deionized water. Gently place the gel into an incubation box. Add Coomassie Brilliant Blue staining solution to cover the gel and gently agitate the solution on a shaking table for 3 hours. Once staining is sufficient, discard the staining solution and wash the gel several times with deionized water. Then, add decolorizing solution to the gel and agitate it until the gel is sufficiently decolorized, repeating this step until clear and visible protein bands are observed.

*1.10 ROS detections in solutions*

Overall ROS Detections: DCFH was employed to detect ROS generation from photosensitizers (PSs) in aqueous solution under white light irradiation. DCFH was obtained by reacting DCFH-DA (0.5 mL, 1 mM in ethanol) with an aqueous NaOH solution (2 mL, 10 mM) for 30 minutes at room temperature. The reaction mixture was then neutralized with 7.5 mL of PBS buffer solution, resulting in a stock solution with a final concentration of 50 μM. The PBS buffer solution containing 50 μM DCFH was mixed separately with different samples. The fluorescence of PS-sensitized DCFH under white light irradiation (20 mW cm^-2^) was measured at various time intervals. The photoluminescence (PL) spectra were recorded with an excitation wavelength of 488 nm, and emission was collected from 500 to 600 nm. The enhancement of DCFH fluorescence intensity (I/I_0_) at 520 nm was used as an indicator of total ROS generation.

Singlet Oxygen (^1^O_2_) Detection: 9,10-Anthracenediyl-bis(methylene) dimalonic acid (ABDA) was used as an indicator to detect the generation of singlet oxygen (^1^O_2_). PBS buffer solution containing 50 μM ABDA (stock solution: 5 mM in DMSO) was mixed separately with different samples, ensuring a final concentration of 50 μM for each sample. The mixture was then irradiated with white light (20 mW cm^-2^), and the absorbance of ABDA at 378 nm was recorded after irradiation. The ^1^O_2_ generation rate was characterized by calculating the decomposition ratio of the absorbance at 378 nm after light irradiation to the initial absorbance (A/A_0_), where A0 represents the initial absorbance and A represents the absorbance after irradiation.

Superoxide Radical (O_2_^•−^) Detection: Dihydrorhodamine 123 (DHR123) was used as an indicator to detect the generation of superoxide anion (O_2_^•−^). PBS buffer solution containing 5 μM DHR123 (stock solution: 2 mM in DMSO) was mixed separately with different samples to achieve a final concentration of 5 μM for each sample. The fluorescence of PS-sensitized DHR123 under white light irradiation (20 mW cm^-2^) was measured at different time intervals. The photoluminescence (PL) spectra were recorded with an excitation wavelength of 488 nm, and emission was collected from 500 to 600 nm. The enhancement of DHR123 fluorescence intensity (I/I_0_) at 527 nm was used as an indicator of total ROS generation.

*1.11 Electron spin resonance (ESR) analysis*

Electron spin resonance (ESR) analysis was conducted to confirm the generation of radicals using DMPO as the spin trap agent. Working samples were prepared by mixing 200 μL of DMSO containing 75 mM DMPO (for O_2_^•−^) or TMPO (for ^1^O_2_) and 1 mM of the photosensitizers (PSs) prior to the analysis. ESR spectra were recorded before and after exposing the samples to white light irradiation (50 mW cm^-2^) for 5 minutes.

*1.12 Cell culture*

MDA-MB-231 and 4T1 cells were cultured in Dulbecco's Modified Eagle Medium (DMEM) supplemented with 10% fetal bovine serum (FBS) and 1% Penicillin/Streptomycin (all purchased from Gibco). The cells were maintained at 37°C in a 5% CO₂ incubator.

*1.13 Cytotoxicity assay*

Cells were seeded at a density of 5 × 10^3^ cells/well into 96-well plates in complete medium. After cell attachment, nanoparticles (NPs) at varying concentrations were added to the wells. Following 24 hours of incubation, 10 μL of MTT solution was added to each well, and the cells were cultured for an additional 2 hours. The absorbance of MTT at 490 nm was measured using a microplate reader (Biotek, VT) to assess cell viability.

*1.14* *Cellular uptake*

MDA-MB-231 cells were digested with trypsin and seeded into confocal fluorescence Petri dishes, with approximately 1 × 10^4^ cells per dish. The dishes were then placed in an incubator. Once the cell density reached 90%, the culture medium was aspirated, and the cells were washed three times with PBS. Fresh medium containing 5 μM **BDPM** or **BDPM@OMVs** was added to the dishes, and the cells were incubated for 0, 15, 30, and 60 minutes, respectively. Hoechst 33342 cytosolic dye was then added to the cells and incubated for 30 minutes. After incubation, the excess dye was washed off with PBS. The cells were then observed under a 63X oil immersion objective using a confocal laser scanning microscope.

*1.15 Intracellular reactive oxygen species detection*

Cells were digested with trypsin and seeded into 6-well plates at a density of approximately 1 × 10⁵ cells per well. The plates were then placed in an incubator. Once the cell density reached 90%, the medium was aspirated, and the cells were washed three times with PBS. The cells were then incubated with the drugs or other treatments for 30 minutes. Following incubation, 10 μM DCFH-DA and 10 μM DHR123 were added to the wells, and the cells were further incubated for 30 minutes. After the incubation, excess dyes were removed by washing the cells with PBS. The samples were then observed using an inverted fluorescence microscope.

*1.16 Live/Dead cell staining assay*

Cells (1 × 103) were seeded into 6-well plates and incubated for 24 hours. The samples were then incubated with nanoparticles (NPs) at a concentration of 100 μg/mL or equivalent treatments for 30 minutes. Following this, the cells were incubated with 5 μM Calcein-AM for 30 minutes to allow for calcium influx detection. Afterward, the cells were washed three times with PBS and incubated with 2.5 μM propidium iodide (PI) for an additional 30 minutes. Finally, the cells were visualized under an inverted fluorescence microscope to assess fluorescence intensity and cell viability.

*1.17 Subcellular Organelle Localization*

MDA-MB-231 cells were digested with trypsin and then seeded into confocal fluorescence Petri dishes, with approximately 1 × 105 cells per dish. The dishes were placed in an incubator. Once the cell density reached 90%, the culture medium was aspirated, and the cells were washed three times with PBS. Next, 5 μM of **BDPM@OMVs** was added for incubation. After 30 minutes, 1 μM of commercial lysosomal green dye (LysoTracker-Green) and 1 μM of mitochondrial green dye (MitoTracker-Green) were added separately to the cells, and incubation continued for an additional 30 minutes. After incubation, excess dye was removed by washing the cells with PBS. The cells were then observed under a confocal microscope to visualize the lysosomal and mitochondrial localization of the dyes.

*1.18 Annexin V/Propidium Iodide (PI) staining*

MDA-MB-231 cells were incubated with **BDPM** or **BDPM@OMVs** for 30 minutes. After irradiation, the treated cells were double-stained with annexin V FITC and propidium iodide (PI) at room temperature for 20 minutes, protected from light. The stained cells were then analyzed using flow cytometry for apoptotic cell quantification and observed under confocal laser scanning microscopy to visualize cell death and apoptosis.

*1.19 Macrophage Polarization*

MDA-MB-231 cells were digested with trypsin and seeded into 6-well plates. Once the cell density reached 80%, 100 ng/mL of interleukin-4 (IL-4) was added to induce M2-type macrophage polarization. After 12 hours of incubation, the cells were washed three times with PBS. Then, 10 μM of **BDPM@OMVs** and 1 μg/mL of lipopolysaccharide (LPS) were added, as per the experimental groups, to induce repolarization of M2-type macrophages to M1-type macrophages. After 12 hours of incubation, the morphological changes of macrophages were observed using an inverted fluorescence microscope.

The cell supernatants were then aspirated and centrifuged at 2000 rpm for 20 minutes. The resulting supernatants were collected for enzyme-linked immunosorbent assay (ELISA) analysis, and the levels of cytokines TNF-α and IL-1β were measured using appropriate ELISA kits.

Simultaneously, the cells were collected using a cell scraper, washed three times with PBS, and centrifuged to remove the supernatant. The cells were resuspended and transferred to EP tubes at a concentration of 1 × 10⁴ cells/100 μL. The cells were then incubated with glucosinolated aldehyde-based erythrocyanin-labeled anti-mouse CD86 antibody (PE-anti-mouse CD86 antibody) for 30 minutes in the dark. Afterward, the cells were washed with PBS and subjected to flow cytometry analysis. The PE fluorescence signal peaks of each group were detected during the flow cytometry experiments.

*1.20* *Construction of the 4T1 hormonal mouse model*

All animal experiments conducted in this study were approved by the Animal Care Committee of Central South University (Changsha, China). Female BALB/c mice, aged 6 weeks, were obtained from the Laboratory Animal Center of Central South University and used to establish the 4T1 tumor-bearing mouse model.

In brief, 1× 10^6^ 4T1 cells were injected subcutaneously into the right flank of the mice to establish the tumor-bearing model. Tumor volume was calculated using the formula: volume 𝐴 = 𝑎 ∗ 𝑏^2^/2, where a represents the length and b represents the width of the tumor. Once the tumor volumes reached approximately 100 mm^3^, the mice were used for subsequent antitumor assays.

*1.21 In vivo PL imaging of tumor-bearing mice*

All animal experiments conducted in this study were approved by the Animal Care Committee of Central South University (Changsha, China). All the animal experiments involved in this work were approved by the animal ethics committee of Central South University (Changsha, China) (license no. XMXH–2023-1384). Female BALB/c mice, aged 6 weeks, were obtained from the Laboratory Animal Center of Central South University and used to establish the 4T1 tumor-bearing mouse model.

Three mice were randomly selected from the tumor-bearing group, anesthetized with isoflurane, and intravenously injected with the drugs via the tail vein. In vivo fluorescence imaging was performed at 0, 12, 24, 48, and 96 hours post-injection, using a small-animal in vivo imaging system. The excitation wavelength was set to 550 nm, and emission was detected in the range of 560-700 nm.

After anesthesia, the mice were imaged sequentially according to the time points corresponding to different experimental groups. The relative fluorescence intensity of the tumors was recorded for each time point. Following in vivo imaging, the mice were euthanized by cervical dislocation. The tumors and major organs (heart, liver, spleen, lungs, and kidneys) were then excised, washed with PBS, and subjected to fluorescence imaging to assess organ-specific distribution.

*1.22 In vivo antitumor effect*

We first evaluated the tumor-suppressive effect of **BDPM@OMVs** in mice with tumors of regular size (initially around 200 mm^3^). The mice were randomly divided into three groups (n = 6), as follows: (i) intratumoral (i.t.) injection of PBS solution (PBS group); (ii) i.t. injection of **BDPM** (**BDPM** group); (iii) i.t. injection of **BDPM@OMVs** (**BDPM@OMVs** group). Tumor volume and body weight were measured on alternate days over a period of 18 days. At the end of the study, tumor tissues were collected and subjected to histological analysis, including H&E staining, TUNEL staining, and Ki67 staining to assess tumor morphology, apoptosis, and cell proliferation, respectively.

**2. Supplemental Figures**


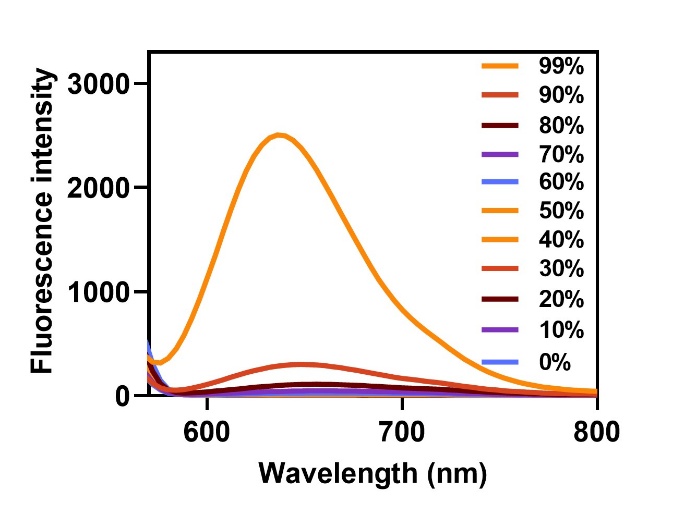


**Figure S1**. Fluorescence spectra of **BDPM** in different PBS/DMSO volume ratios.


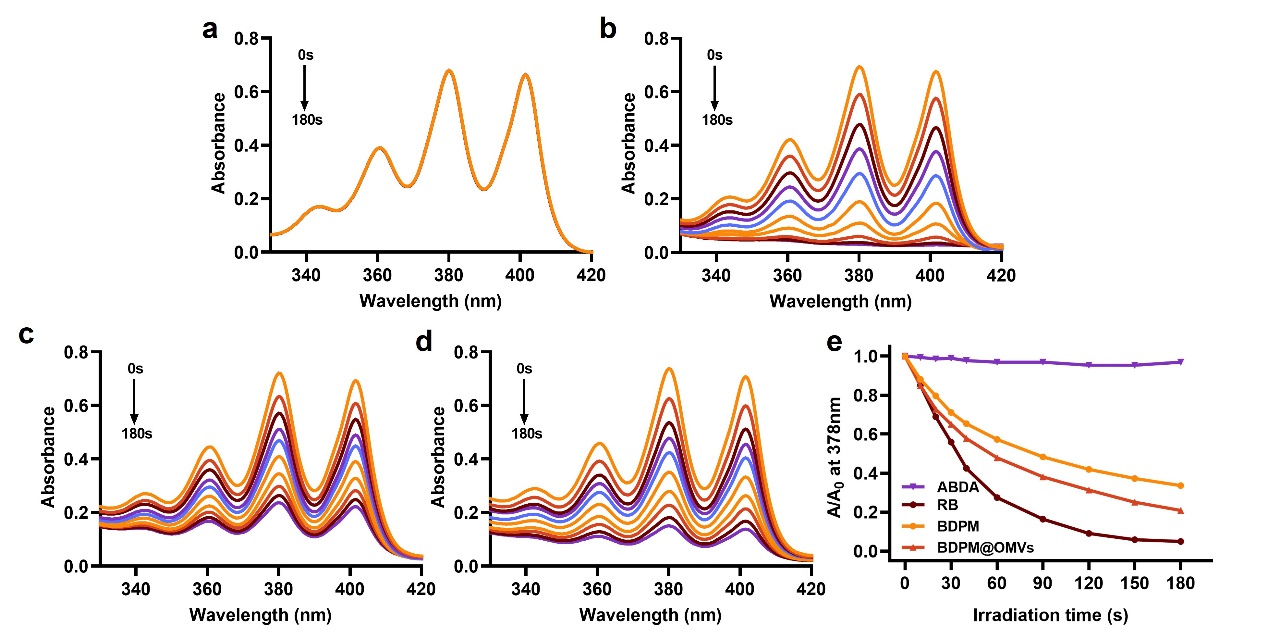


**Figure S2**. Absorbance spectra of ABDA (50 µM) in the presence of (a) **PBS**, (b) **RB**, (c) **BDPM,** (d) **BDPM@OMVs** under different irradiation time. The concentration of PSs was 10 µM.


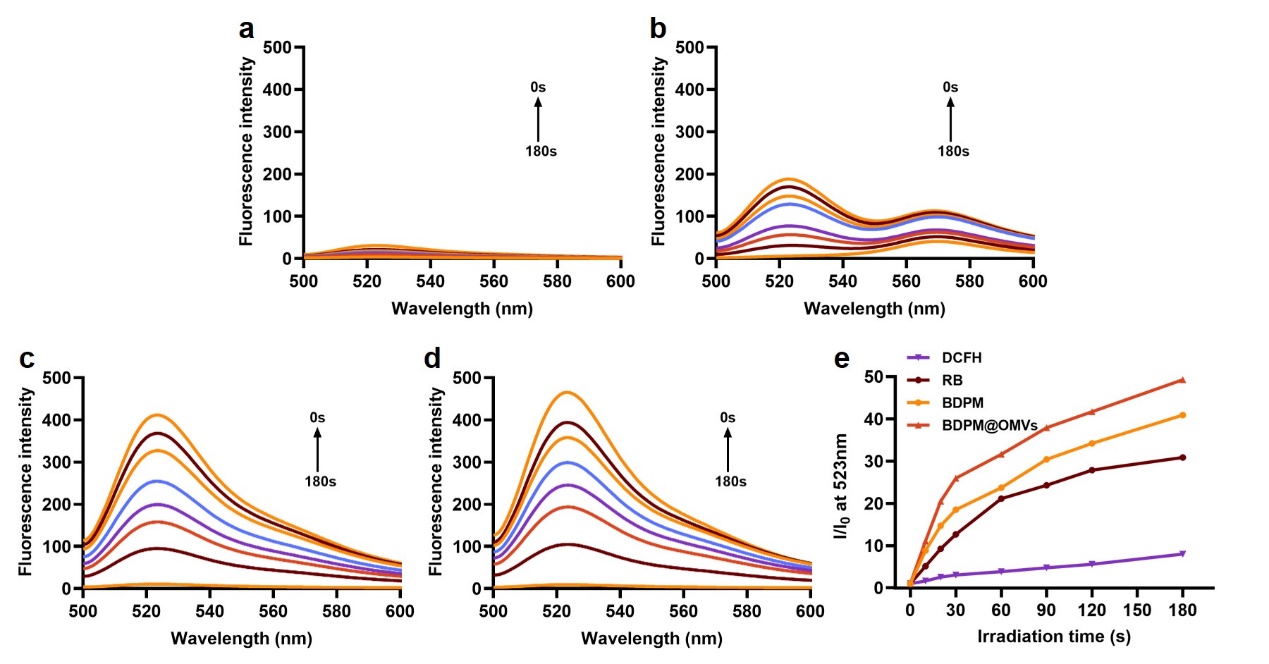


**Figure S3.** Fluorescence spectrum of DCFH (50 µM) in the presence of (a) **PBS**, (b) **RB**, (c) **BDPM**, (d) **BDPM@OMVs** under different irradiation time. The concentration of PSs was 10 µM. Em = 480 nm.


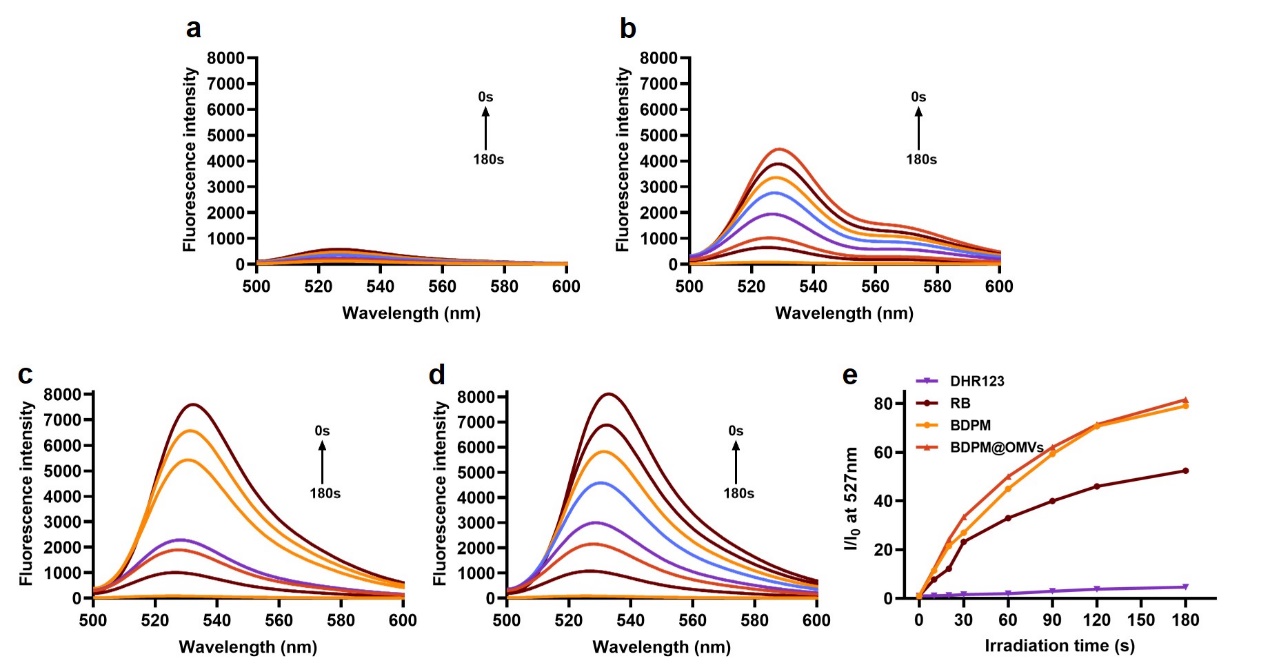


**Figure S4.** Fluorescence spectrum of DHR123 (5 µM) in the presence of (a) **PBS**, (b) **RB**, (c) **BDPM**, (d) **BDPM@OMVs** under different irradiation time. The concentration of PSs was 10 µM. Em = 480 nm.


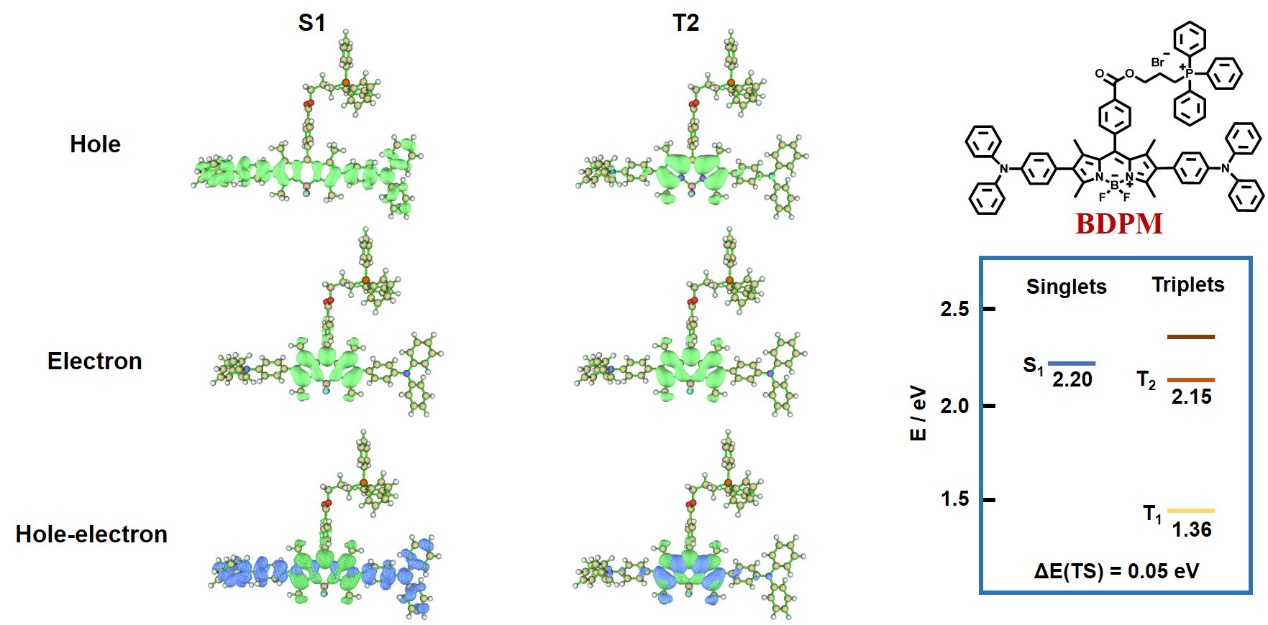


**Figure S5.** Electron-hole orbital analysis of **BDPM**.


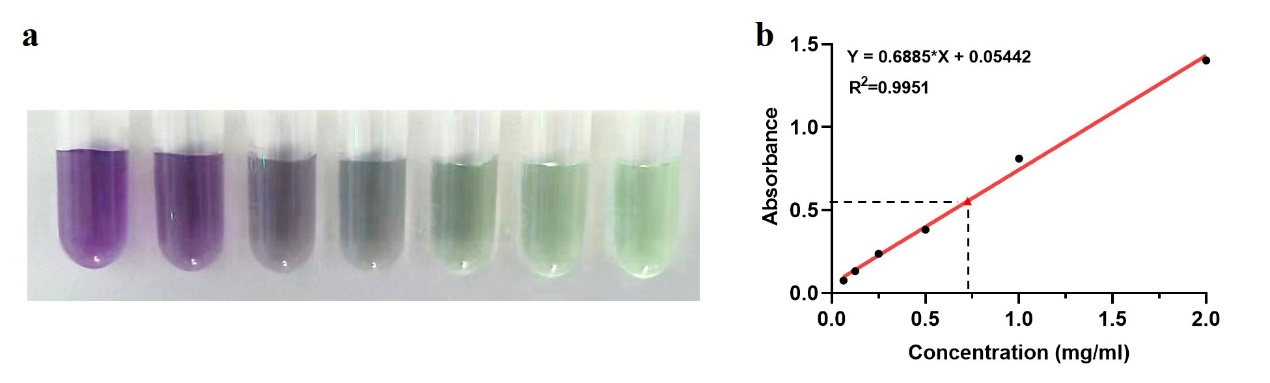


**Figure S6.** Quantitative protein analysis of OMVs. a. Physical drawing of BCA kit standard. b. Concentration of OMVs obtained under the standard curve of BCA kit.


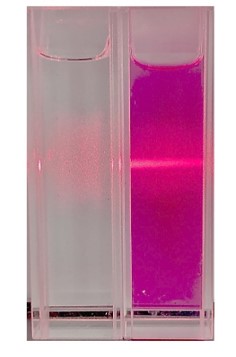


**Figure S7.** Tyndall phenomenon presented under red laser pointer irradiation, left side: **PBS**, right side: **BDPM@OMVs**.


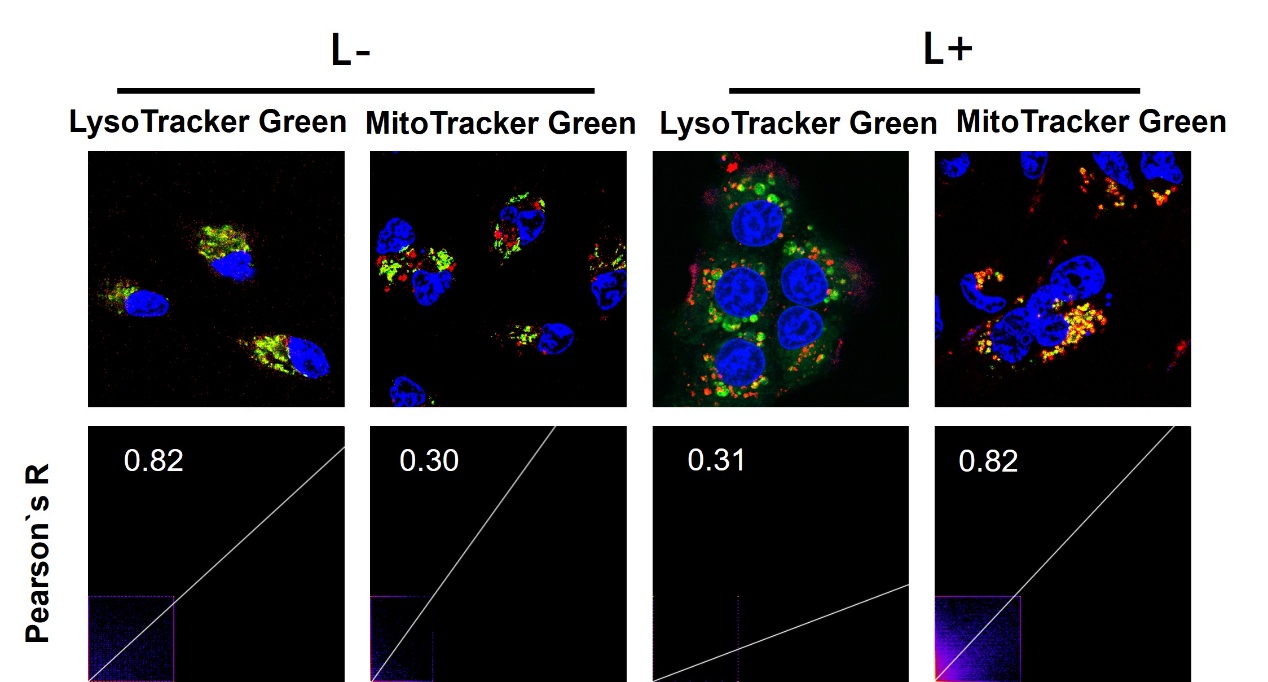


**Figure S8.** Colocalization images of MDA-MB-231 cells co-stained with **BDPM@OMVs** and LysoTracker Green/MitoTracker Greenb (Scale bar: 10 μm).


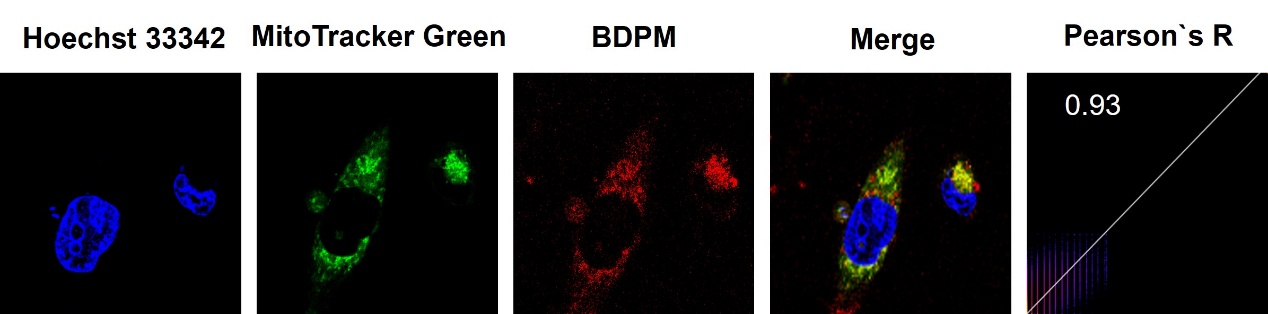


**Figure S9.** Colocalization images of MDA-MB-231 cells co-stained with **BDPM** and MitoTracker Green (Scale bar: 10 μm).


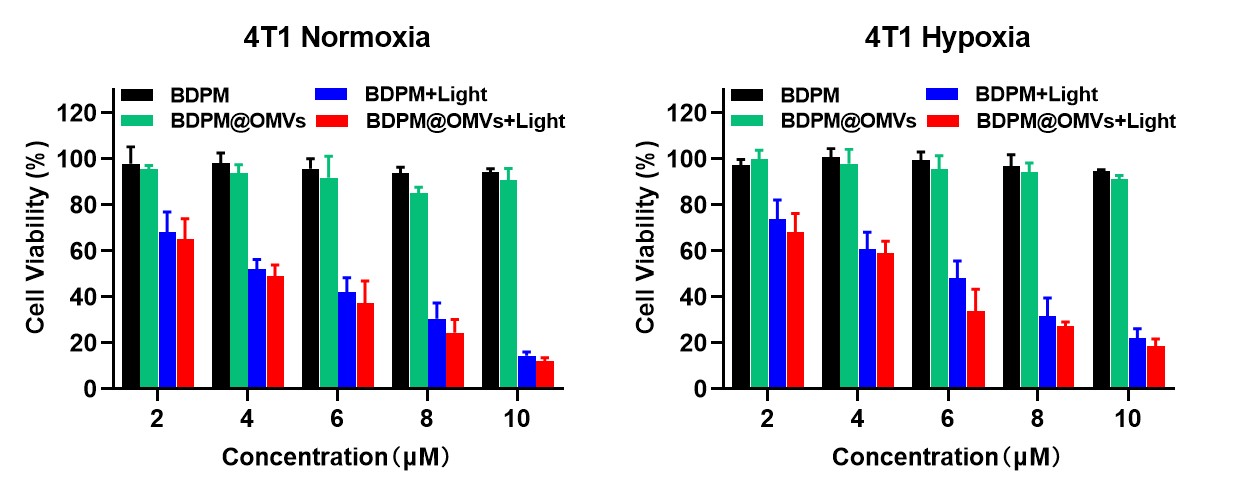


**Figure S10.** Relative viability of 4T1 cells after 24 h co-incubation with **BDPM** and **BDPM@OMVs** under darkness and irradiation in normoxic condition and hypoxic condition. Data are shown as mean ± SD (n = 3).


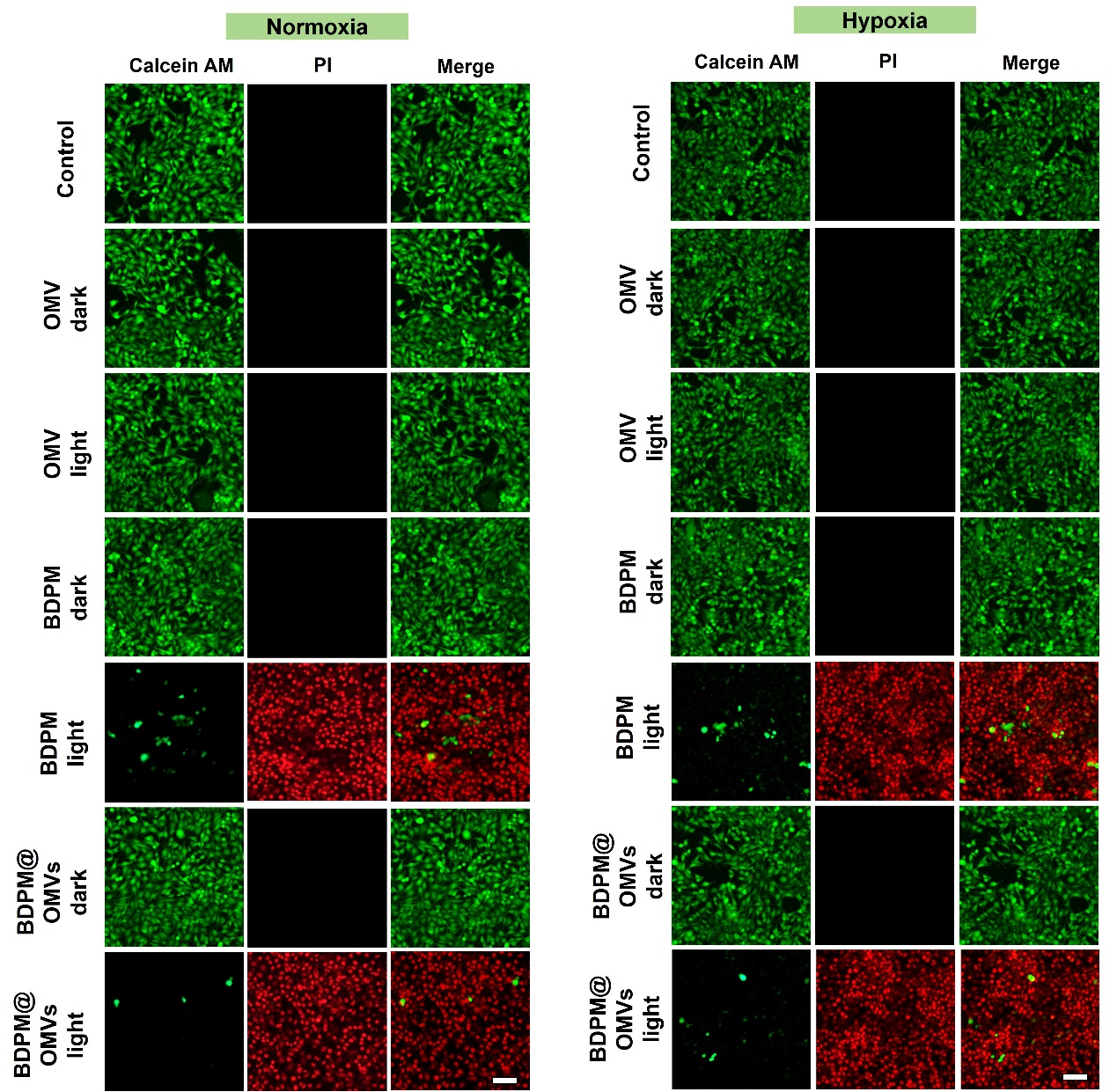


**Figure S11.** Fluorescent images of MDA-MB-231 cells after various treatments as indicated. The cells are stained with Calcein-AM for live cells and propidium iodide (PI) for dead cells (Scale bar: 200 μm).


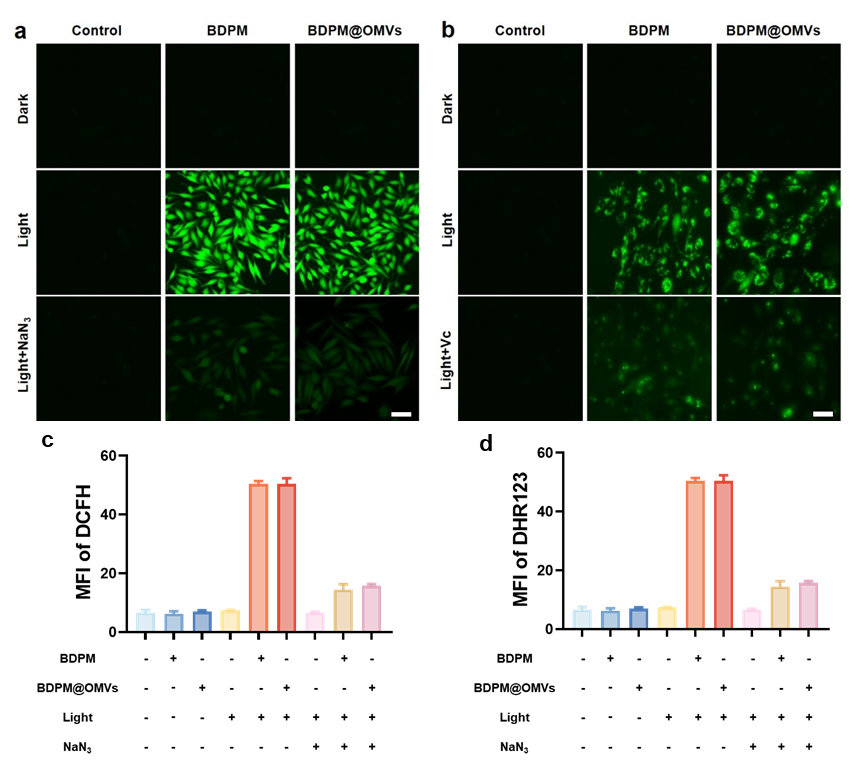


**Figure S12.** Fluorescence images of intracellular ROS generation stained by DCFH-DA and DHR123 under various treatments (Scale bar: 20 μm).


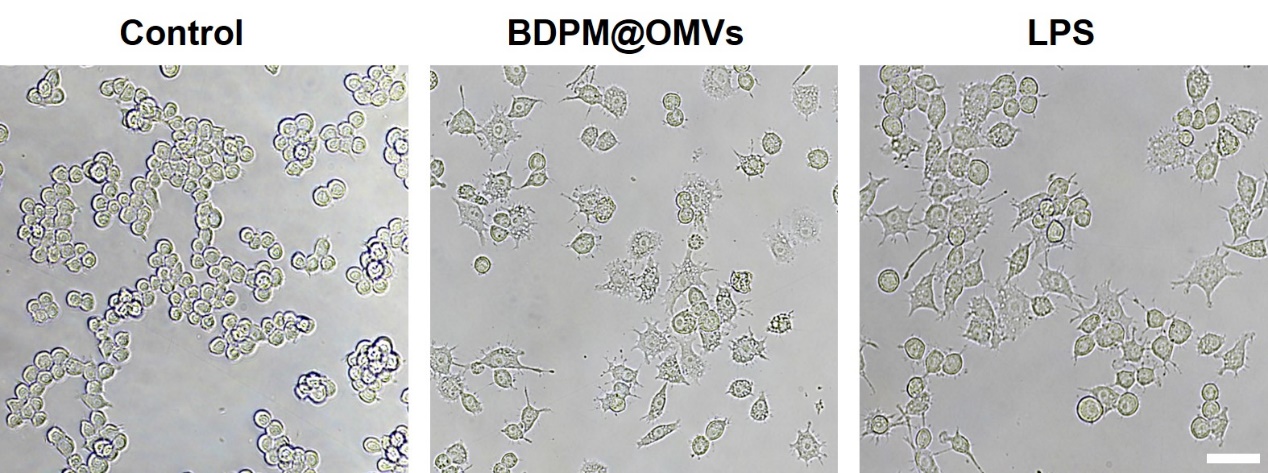


**Figure S13.** Bright field of RAW264.7 cells pretreated with IL-4 after 12 h incubation with **BDPM@OMVs** or LPS, respectively (Scale bar: 50 μm).


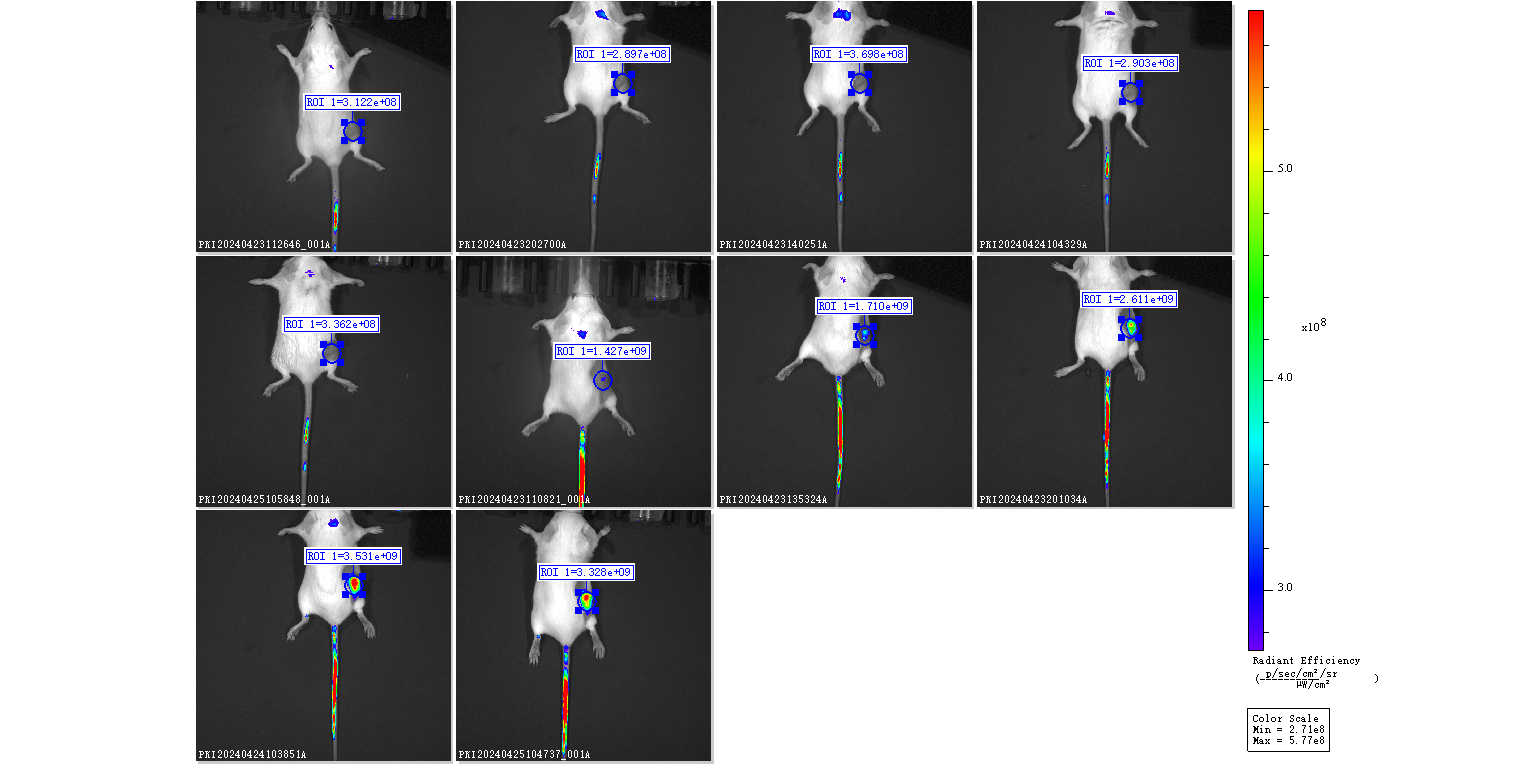


**Figure S14**. Quantitative fluorescence intensity analysis chart for small animal imagers.


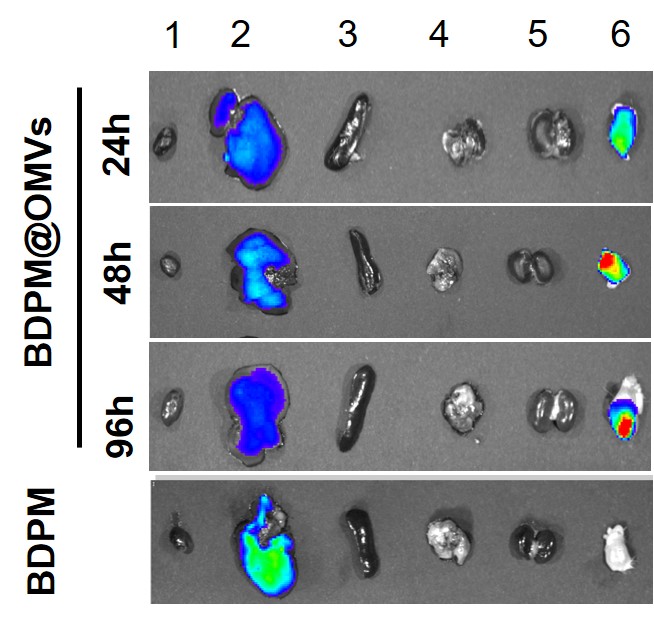


**Figure S15**. The bio-distribution of **BDPM@OMVs** in mice major organs and tumors. The 4T1 tumor-bearing mice were injected with **BDPM** or **BDPM@OMVs** via the tail vein. The mice were euthanized on 24h, 48h and 96h. The major organs, including the heart (1), liver (2), spleen (3), lungs (4), and kidneys (5) and tumors (6) were collected.


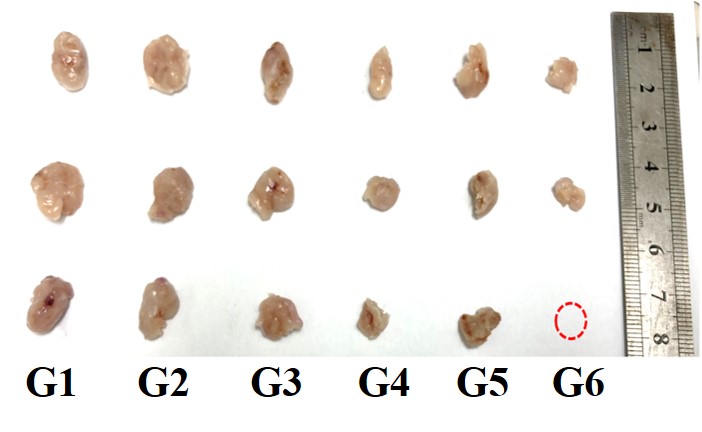


**Figure S16**. Photos of tumors after treatment. Control (G1), **BDPM** (G2), **BDPM@OMVs** (G3), **Light** (G4), **BDPM+Light** (G5), **BDPM@OMVs+Light** (G6).


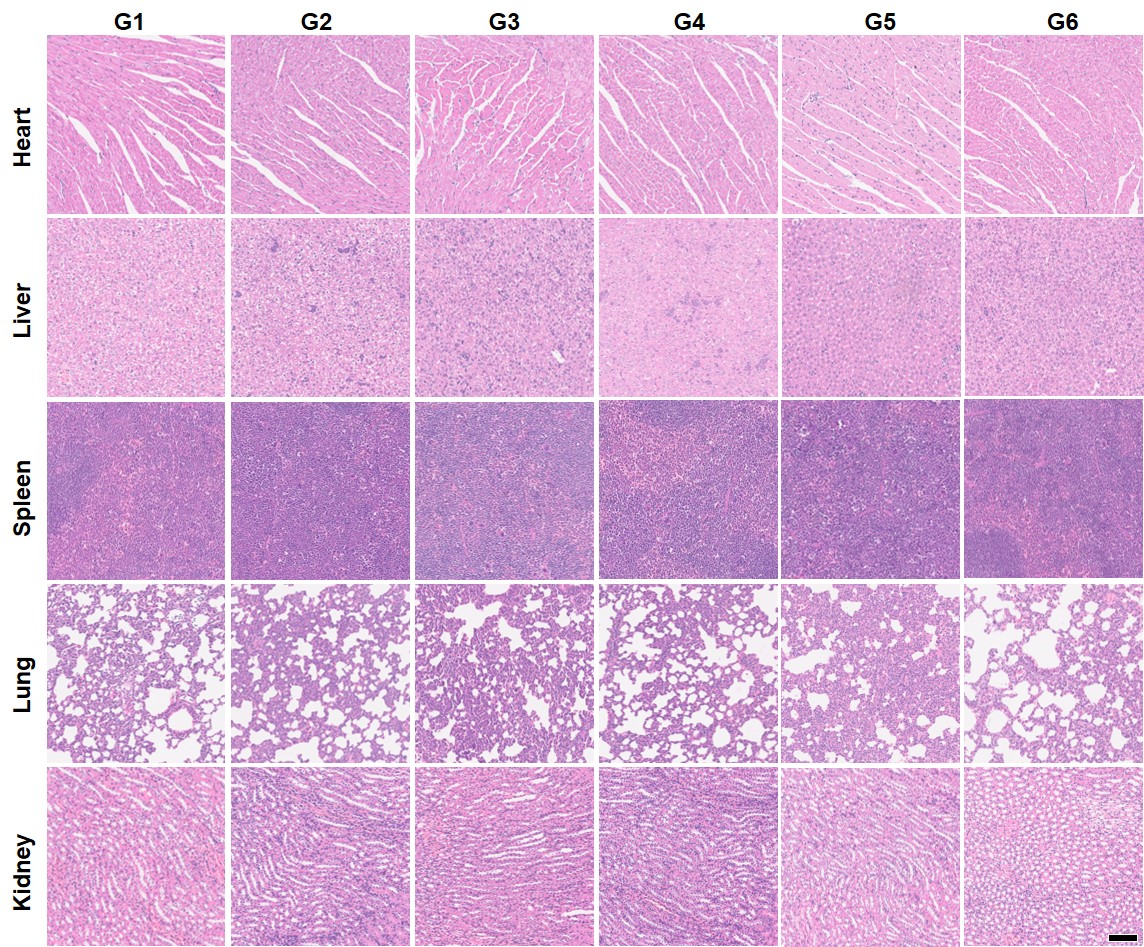


**Figure S17**. H&E staining of major organ tissues in different groups of 4T1 hormonal mice. **Control** (G1), **BDPM** (G2), **BDPM@OMVs** (G3), **Light** (G4), **BDPM+Light** (G5), **BDPM@OMVs+Light** (G6) (Scale bar: 50 μm).


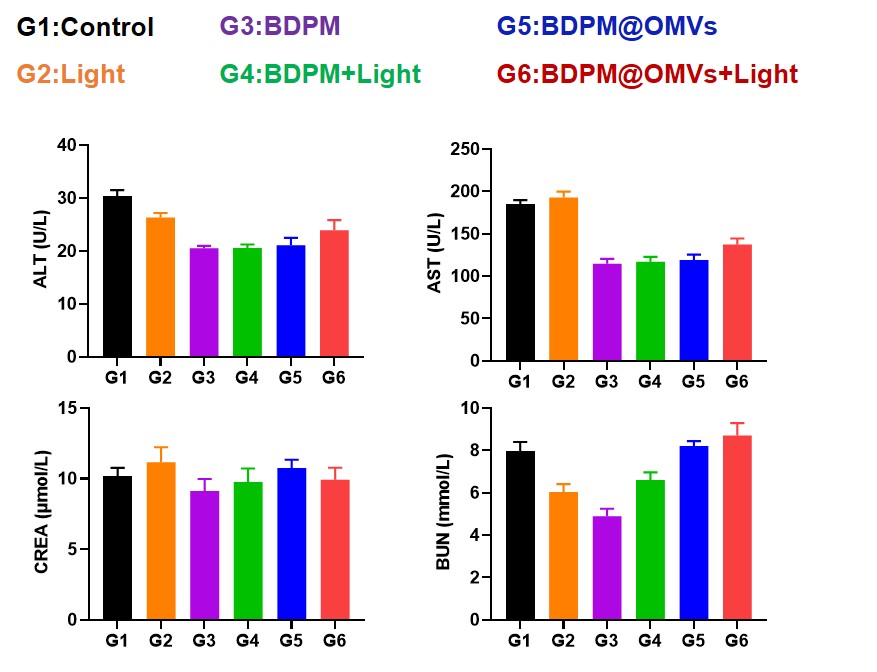


**Figure S18**. Blood biochemistry (ALT, AST, CREA and BUN) was assayed in different groups of 4T1 hormonal mice. Data are shown as mean ± SD (n = 3).


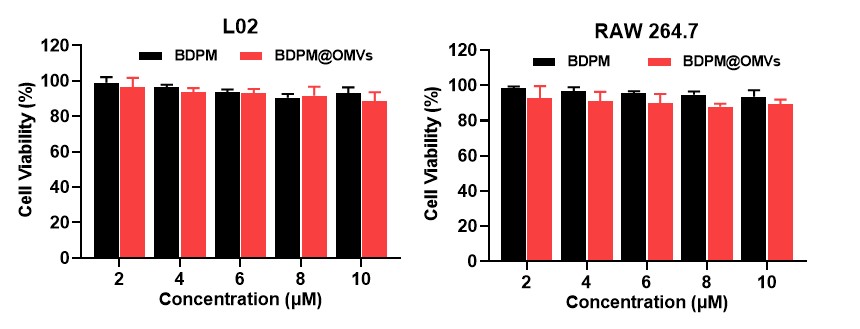


**Figure S19**. Cell viability of L02 cells and RAW264.7 cells after incubation with different concentrations of **BDPM** and **BDPM@OMVs**. Data are shown as mean ± SD (n = 3).


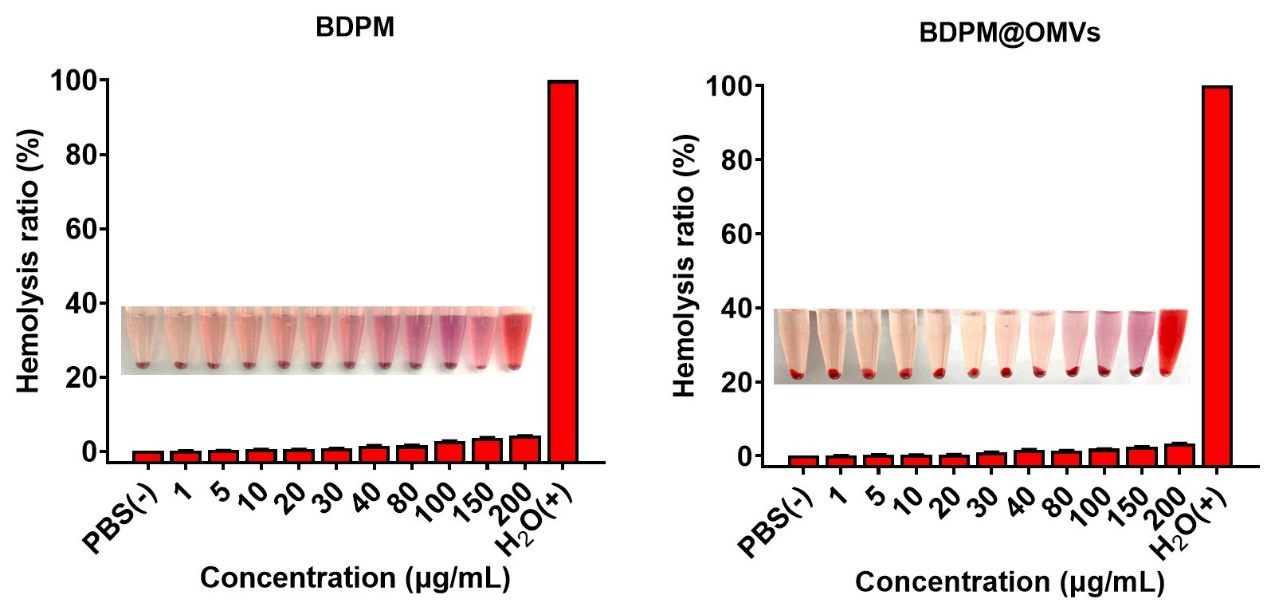


**Figure S20.** Hemolytic evaluation of different concentrations of **BDPM** and **BDPM@OMVs.** Data are shown as mean ± SD (n = 3).


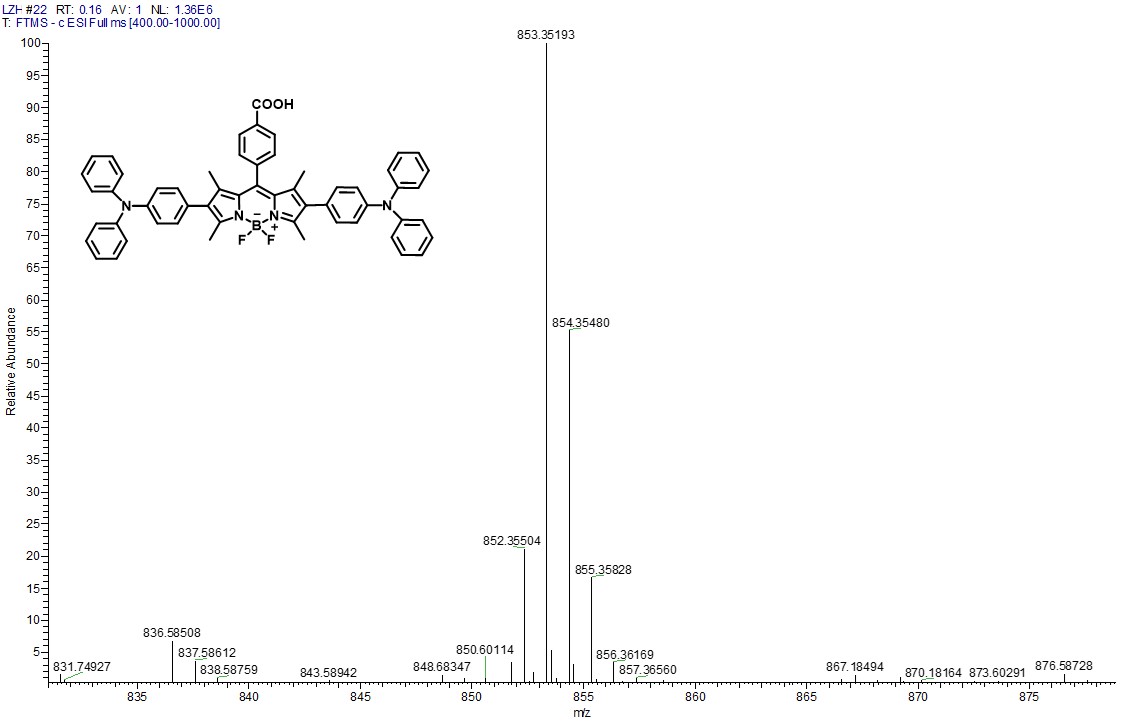


**Figure S21.** HRMS spectra of compound **3**.


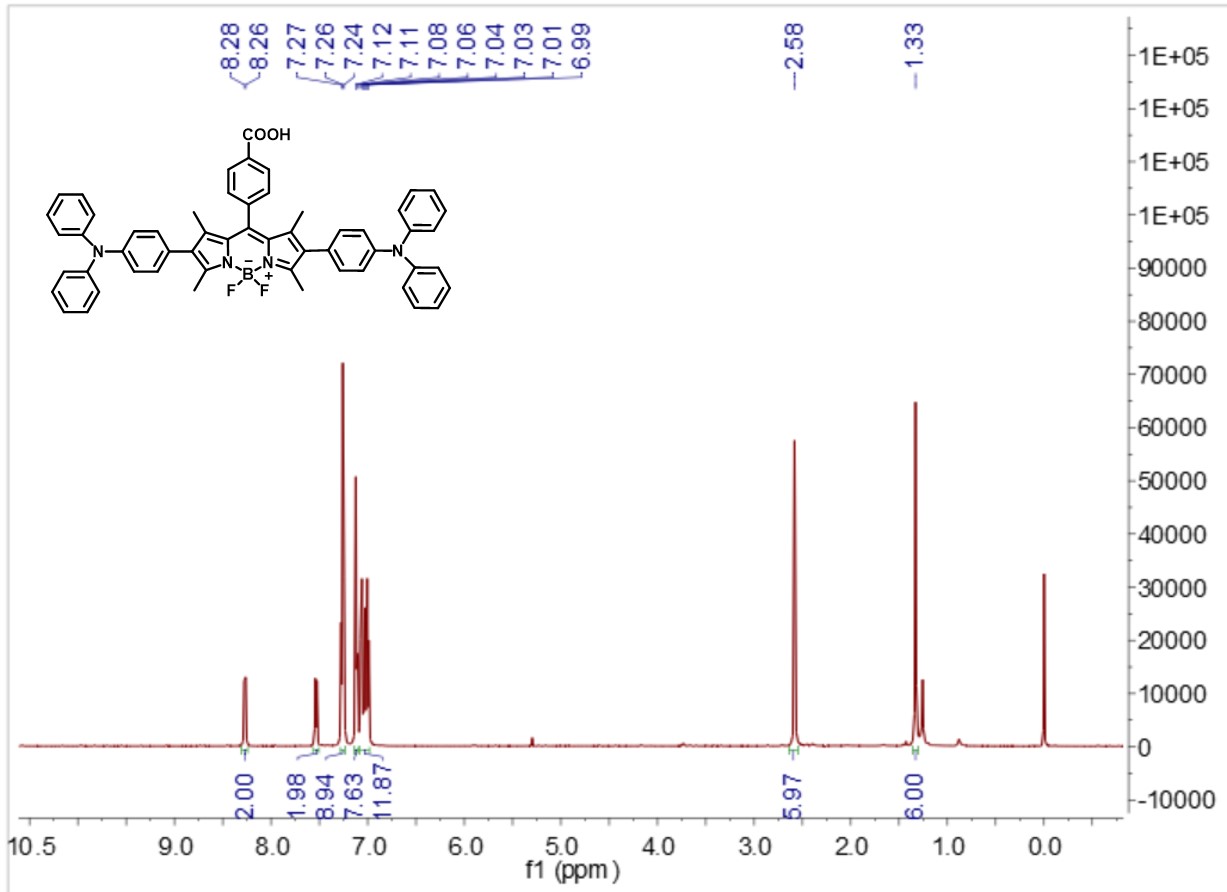


**Figure S22.** ^1^H NMR spectra of **3** in CDCl_3_.


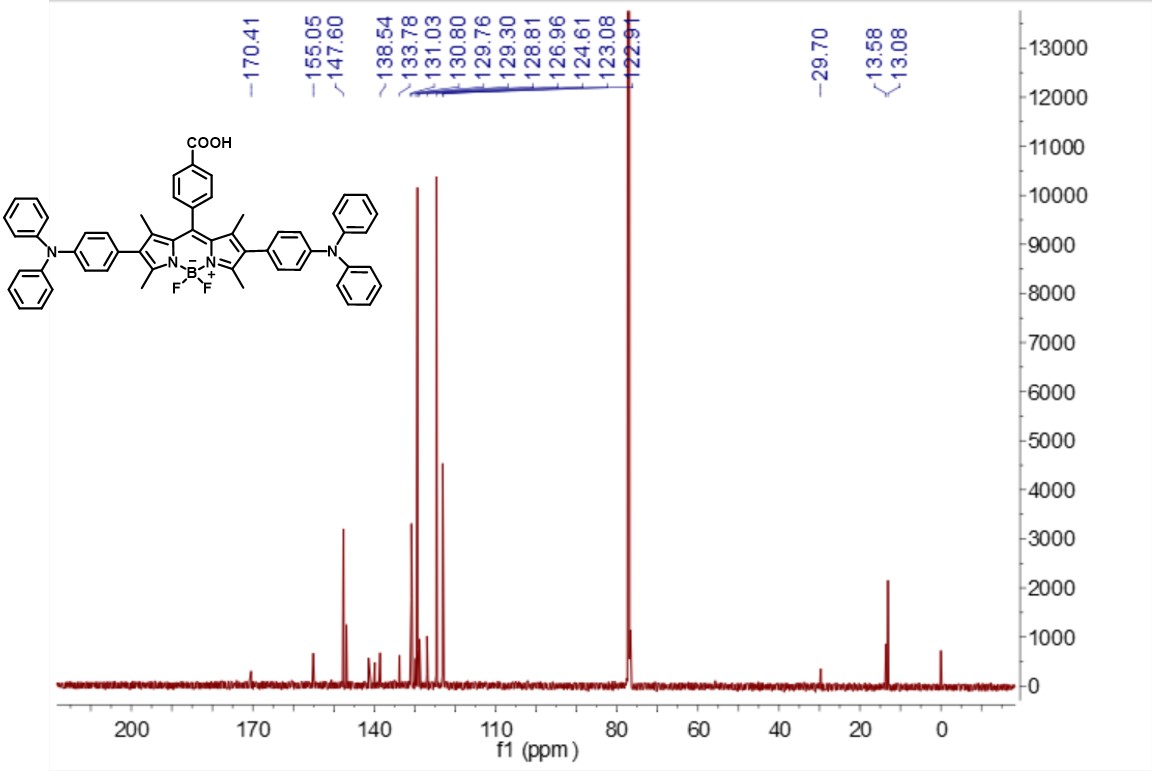


**Figure S23.** ^13^C NMR spectra of **3** in CDCl_3_.


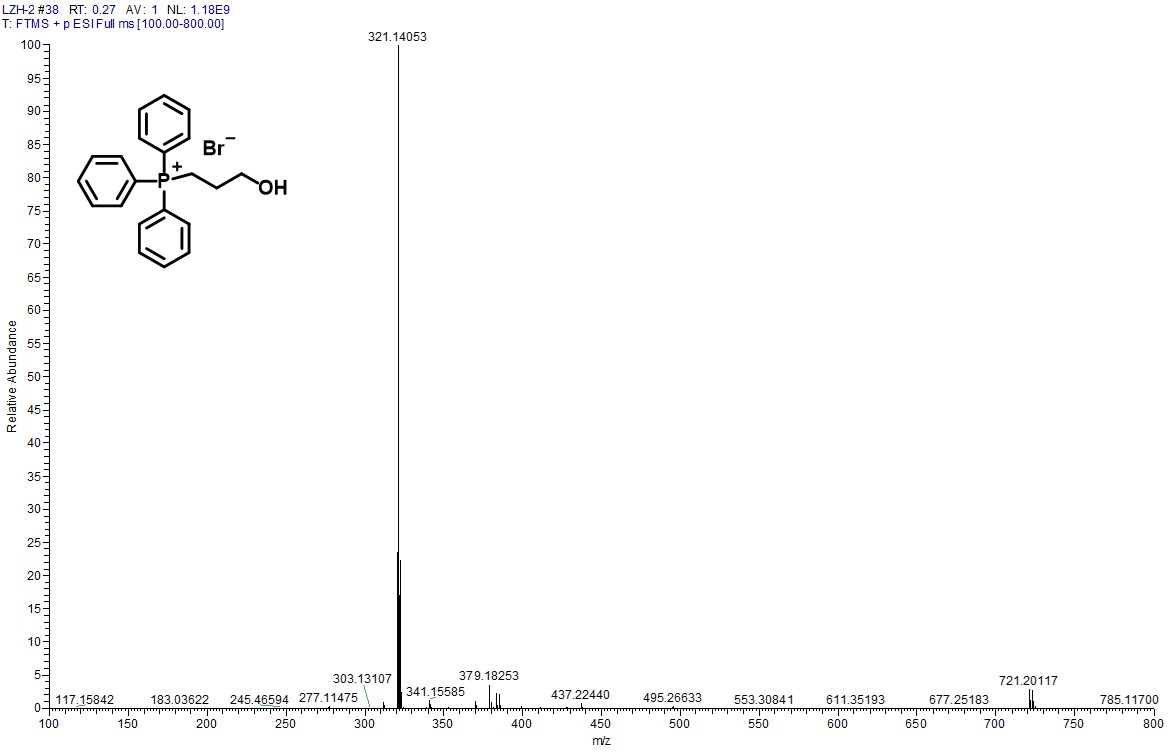


**Figure S24.** HRMS spectra of compound **M-1**.


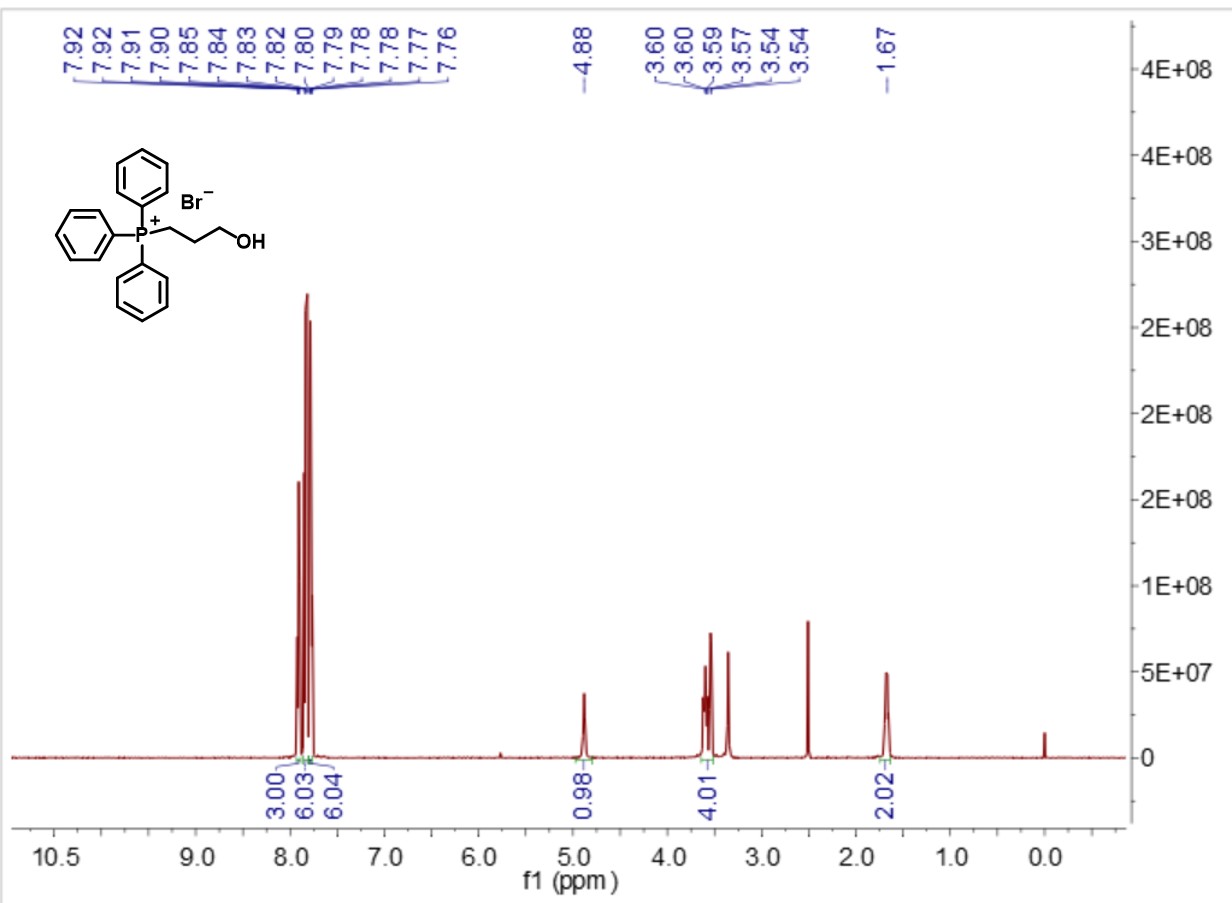
 **Figure S25.** ^1^H NMR spectra of **M-1** in DMSO-*d6*.


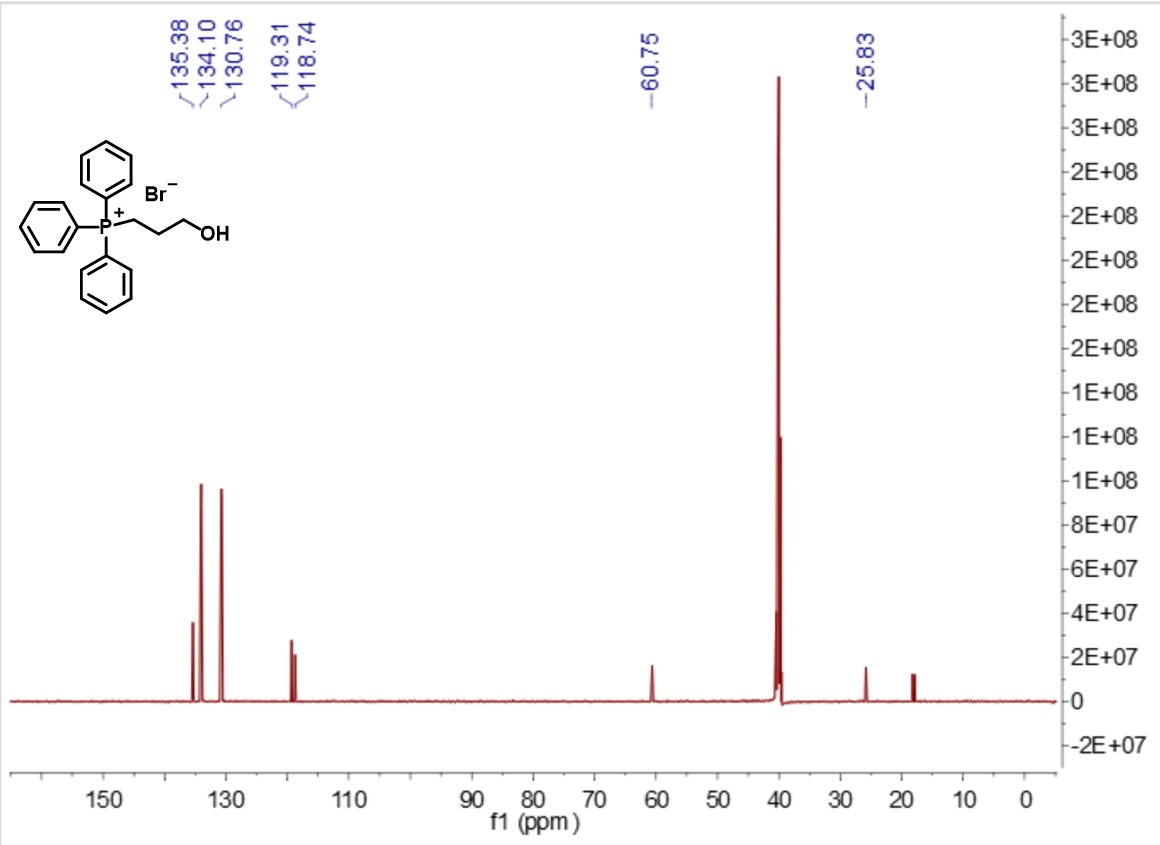
 **Figure S26.** ^13^C NMR spectra of **M-1** in DMSO-*d6*.


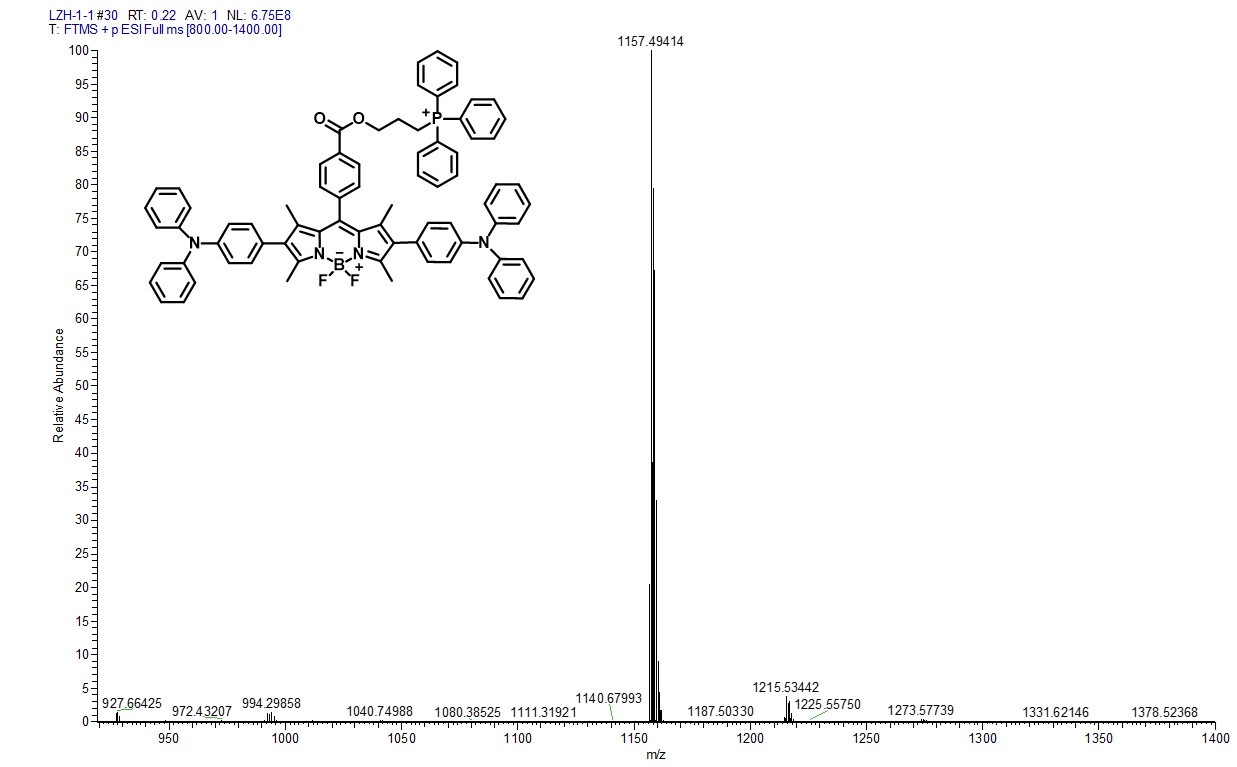
 **Figure S27.** HRMS spectra of compound **BDPM**.


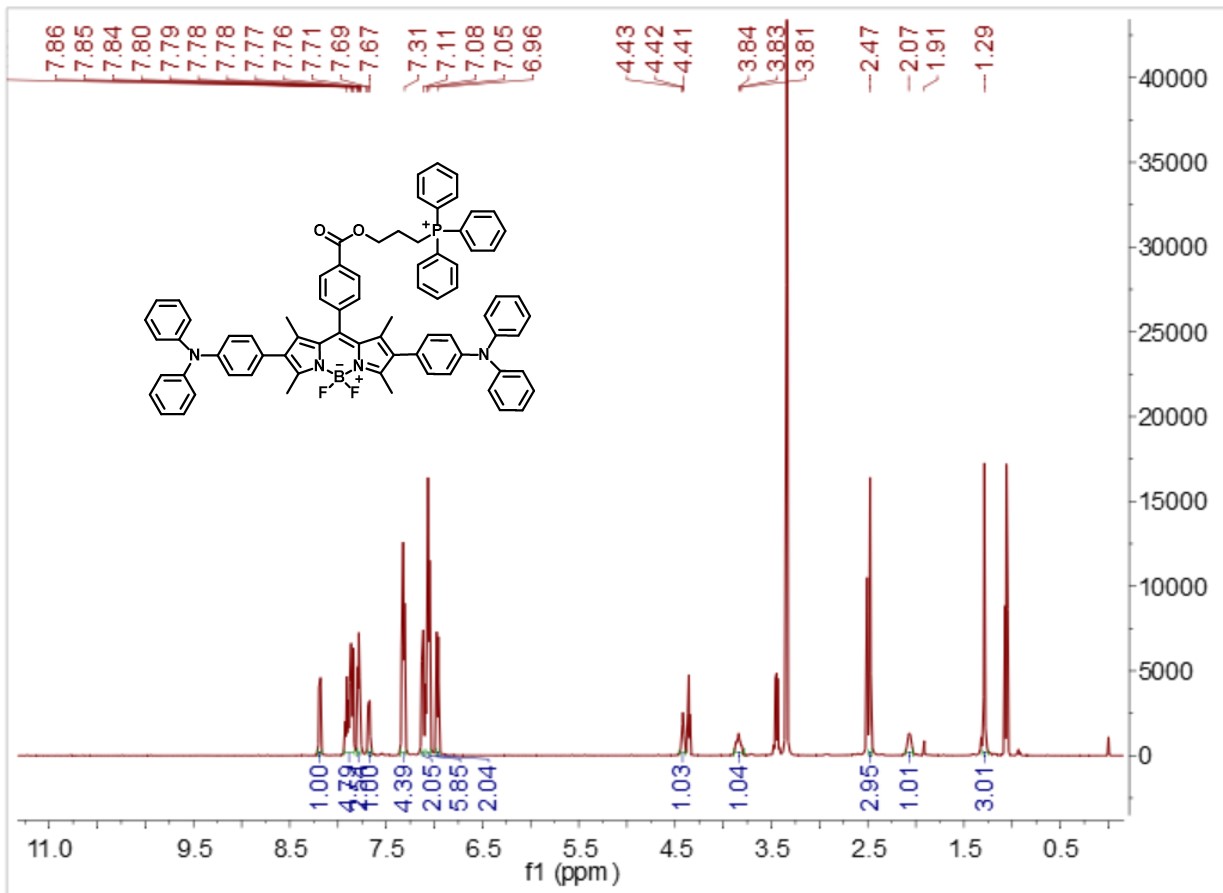
 **Figure S28.** ^1^H NMR spectra of **BDPM** in DMSO-*d6*.


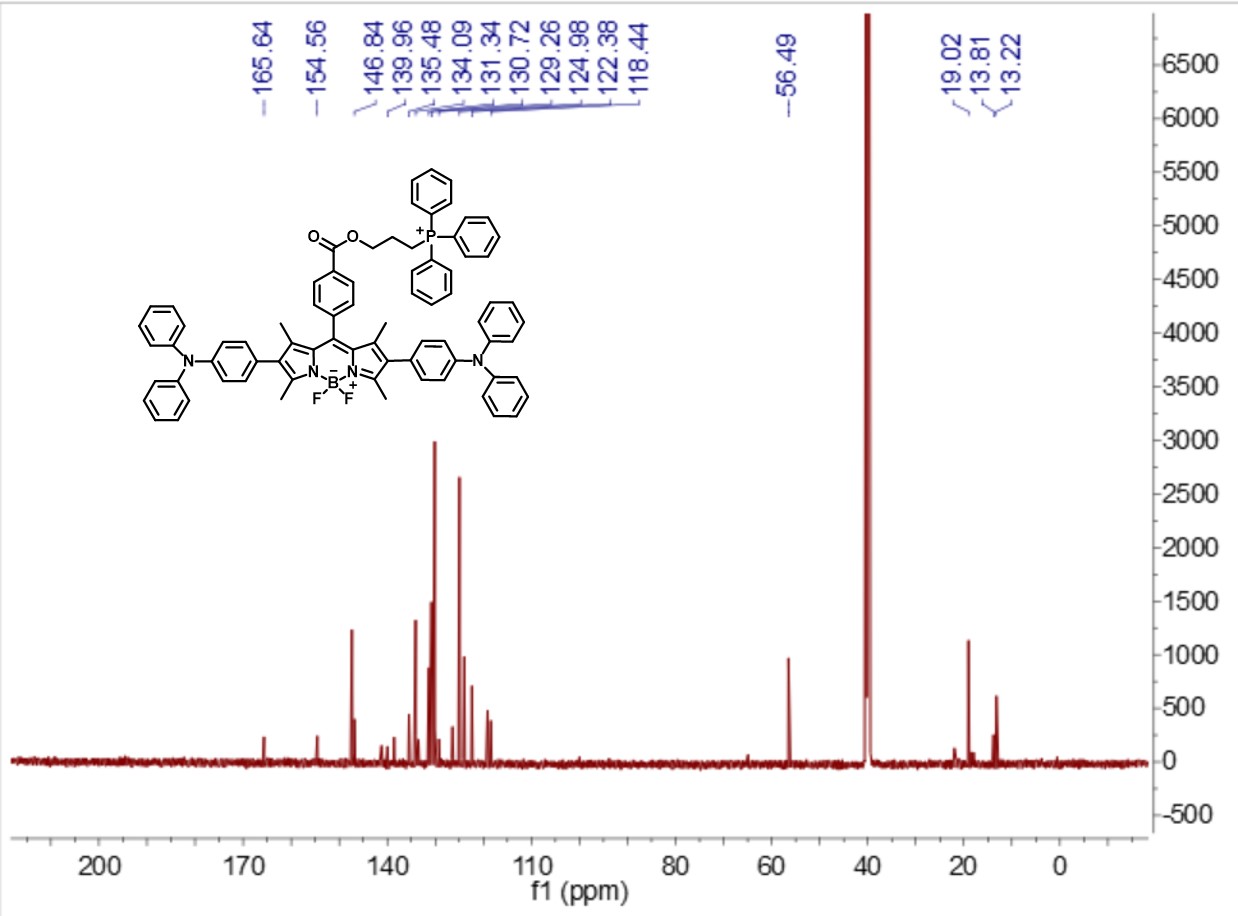
 **Figure S29.** ^13^C NMR spectra of **BDPM** in DMSO-d6.
